# Polycyclic Hydrocarbons from [4*n*]Annulenes: Correlation versus Hybridization Forces in the Formation of Diradicaloids

**DOI:** 10.1002/anie.202209138

**Published:** 2022-09-14

**Authors:** Sergio Moles Quintero, Michael M. Haley, Miklos Kertesz, Juan Casado

**Affiliations:** ^1^ Department of Physical Chemistry University of Málaga 29071 Málaga Spain; ^2^ Department of Chemistry & Biochemistry and Materials Science Institute University of Oregon Eugene OR 97403-1253 USA; ^3^ Department of Chemistry and Institute of Soft Matter Georgetown University Washington DC 20057-1227 USA

**Keywords:** annulenes, antiaromaticity, diradical character, electron correlation, hybridization energy

## Abstract

The conceptual connections between [4*n*] Hückel antiaromaticity, disjoint orbitals, correlation energy, pro‐aromaticity and diradical character for a variety of extended π‐conjugated systems, including some salient recent examples of nanographenes and polycyclic aromatic radicals, are provided based on their [4*n*]annulene peripheries. The realization of such structure–property relationships has led to a beneficial pedagogic exercise establishing design guidelines for diradicaloids. The antiaromatic fingerprint of the [4*n*]annulene peripheries upon orbital interactions due to internal covalent connectors gives insights into the diradicaloid property of a diversity of π‐conjugated molecules that have fascinated chemists recently.

## Graphical abstract

A conceptual electronic structure trip from [4*n*] Hückel antiaromatics to diradicaloids and to more hierarchical complex molecular shapes made by internal covalent hybridization of the peripheral [4*n*] π‐electron circuit is described. Focus is on the underlying physical organic chemistry implications.

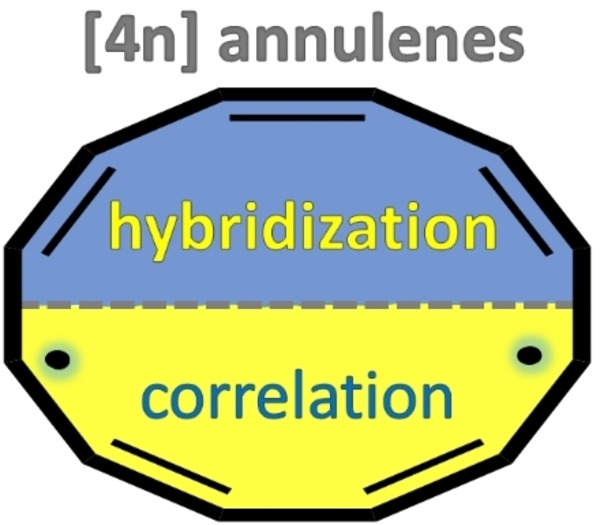

## 1 Introduction

Diradicaloid molecules[^1^, ^2^, ^3^, ^4^] represent cornerstone systems in which the nature of the chemical bond and the validity of its theories can be explored. Recently, these systems have also gained relevance as multipurpose substrates for organic electronic applications.[^5^, ^6^, ^7^, ^8^] There are several approaches for their design, three of which are of interest here: (i) diradicaloids made of non‐alternant antiaromatic [4*n*]annulene hydrocarbons, (ii) those developed from quinoidal cores (or pro‐aromatic molecules in the sense that they are prone to form aromatic structures by homolytic bond rupture), and (iii) those made of a combination of (i) and (ii). In this article the term [4*n*]annulene is used in two different ways: in [*n*]annulene, n is the total number of carbon atoms in the ring, and in [4*n*]annulene, *n* refers to the Hückel's rule definition for an antiaromatic ring.

The recent literature on the diradicaloid topic is replete with a large diversity of examples[^9^, ^10^, ^11^, ^12^] wherein, however, more detailed explanations of the origin of the mosaic of diradicaloid properties would be desirable. To attempt this, we review the basic concepts that give rise to the emergence of the diradical character in the important area of polycyclic hydrocarbon compounds based on [4*n*]annulene peripheries, with particular emphasis in those which are internally “functionalized” or “hybridized” by covalent structures. We will address structure–diradical relationships by analyzing the orbital structures of the [4*n*] π‐electron circuit interpreted in terms of two energy contributions, disjoint diradical correlation energy (*E*_corr_, due to electron–electron repulsion) and hybridization energy (*E*_hybr_, due to connecting bonds). The starting point of this qualitative discussion are the disjointed π‐electron frontier orbitals of [4*n*]annulenes, which are then perturbed by the internally connecting covalent bonds. Orbital hybridization in this case refers to the interactions generated by these internal covalent connectors, which are indicated by red dashed and continuous lines in the figures below. This approach has been already considered elsewhere.[^13^, ^14^, ^15^, ^16^]

**Scheme 1:**
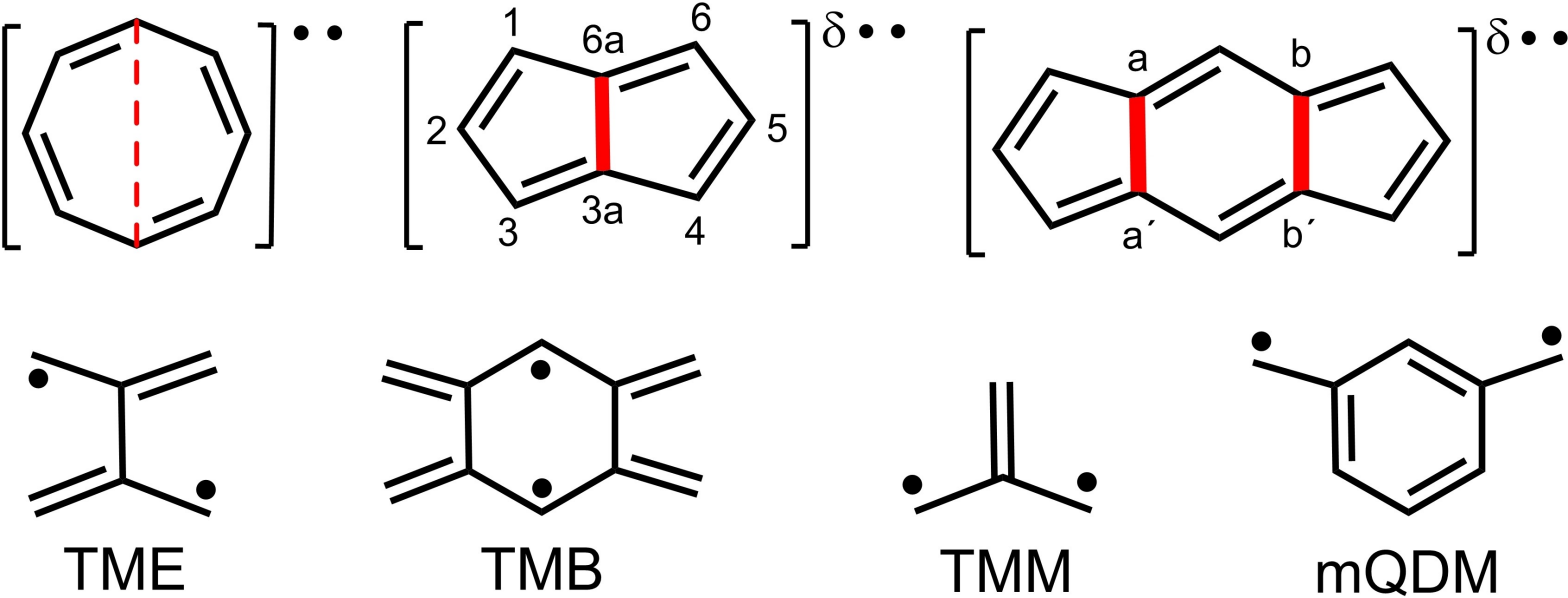
Top: planar *D*_8*h*_ cyclooctatetraene, COT, pentalene and *s*‐indacene (from the left). δ•• denotes diradicaloid character. Covalent connectors are in red. Bottom: pro‐diradicaloid fragments.

The article begins with pentalene and subsequently extends the discussion to *s*‐indacene shown in Scheme 1 as the first representative cases of (i) and (iii) based on the *n*=2 cyclooctatetraene, COT. The [4*n*]annulene case with *n*=3 is reviewed with special focus given the rich family of antiaromatic molecules derived from it,^[17]^ such as heptalene, biphenylene, and their π‐extended derivatives. Then, we move to the odd‐electron benzene‐based phenalenyl radical, in order to explore the antiaromatic fingerprint in connection with the diradical character in polycyclic conjugated systems based on benzenoid molecules. This will give way to address the larger members of the phenalenyl family such as olympicene and triangulenes. With similar arguments, rhombenes as well as long acenes, all being formally described as [4*n*+2]annulene analogues of our [4*n*] antiaromatics, will also be addressed. In these discussions, the connection of the electronic structure will be linked with the description of their main spectroscopic properties and their organic electronics applications.

The analysis of these molecules under the connection of the electronic structure of [4*n*] antiaromatic fragments and their diradicaloid character will permit the general reader to conceptualize and recognize the underlying design rules present in a large variety of polycyclic hydrocarbon diradicaloids of very different topologies. The trip from textbook Hückel theory of [4*n*] antiaromatic systems up to the description of their unique spectroscopic properties and of their applications will serve chemists of different disciplines to appreciate the relevance and bright future of these [4*n*]annulene‐based diradicaloids.

We accompany the present Minireview with a Supporting Information file containing new calculations as well as some definitions that need extended explanations.

## 2 Connection of [4n]Annulene Properties and Diradical Character: Pentalene and Derivatives

**Figure 1:**
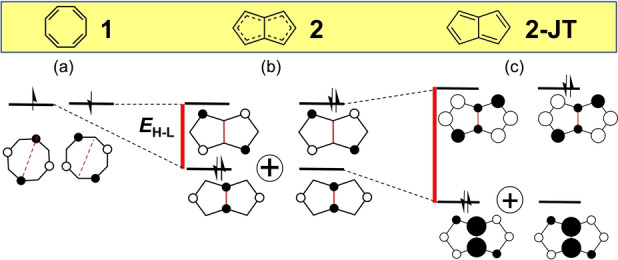
Evolution of the frontier molecular orbitals from planar COT **1** (a) to *D*_2*h*_ pentalene (b, diradicaloid) and to *C*_2*h*_ pentalene (c, diradicaloid including the second‐order Jahn–Teller distortion). The doubly excited H,H→L,L configurational mixing refers to the sum of two configurations. Red solid lines denote the *E*_H–L_ from the hybridization effect, see text. Red broken lines mark the connecting atoms. The sizes of the orbital coefficients are qualitative.

The π‐bonding in pentalene emerges from planar *D*_8*h*_ cyclooctatetraene (COT, **1** in Figure 1), an [8]annulene antiaromatic hydrocarbon, by adding a covalent connector indicated by a red dashed line. An essential starting point of our discussion is that *D*_8*h*_ COT is a Hückel diradical due to several factors: it has two *degenerate* frontier orbitals that are occupied by one electron each (i.e., SOMO, singly occupied molecular orbital) such as shown in Figure 1a. These two frontier orbitals are also of *non‐bonding* character (each atom with non‐vanishing atomic orbital coefficients is surrounded by vicinal atoms which are wavefunction nodes) and of a *disjoint* nature[^1^, ^2^, ^4^] (each orbital has wavefunction contributions on different sets of alternating atoms). This is critical in the sense that the placement of individual electrons in disjoint orbitals largely minimizes the inter‐orbital electronic repulsion energy [i.e., *E*(•/•) or Coulomb repulsion energy between electrons 1 and 2 in the two different SOMO orbitals, commonly denoted as *J*_12_] due to the reduced overlap by virtue of no common π‐orbital coefficients on the same atoms. In contrast, the situation of double occupancy of one of these degenerate orbitals would substantially increase the intra‐orbital electronic repulsion [i.e., *E*(••), or Coulomb repulsion energy between electrons 1 and 2 in the same orbital]. Hence, one can define the disjoint diradical correlation energy as *E*_corr_(disjoint)=*E*(•/•)−*E*(••),&ek meaning that *E*_corr_ estimated between disjoint orbitals would be generally negative and thus favoring the open‐shell diradical configuration. Hence, *E*_corr_ ensures planar COT (*D*_8*h*_) to be a diradical;^[18]^ see Section 1 in the Supporting Information for further details.

Once internal bonds are introduced to transition from COT to pentalene (**2**), connecting the two opposite C(3a) and C(6a) carbon atoms shown in Scheme 1 and Figure 1, the diradical character disappears in Hückel theory even at the high symmetry geometry (*D*_2*h*_). The new C(3a)−C(6a) internal bond produces a *D*_2*h*_ structure with a bonding stabilization of one of the two COT degenerate orbitals that transforms into the HOMO of pentalene, whereas the other orbital remains essentially unaltered and becomes the LUMO. This lifting of the frontier orbital degeneracy of COT gives way to a HOMO–LUMO energy gap in pentalene, leaving, however, the disjoint property of its LUMO intact (Figure 1b). This HOMO–LUMO gap (*E*_H–L_) is caused by the orbital mixing between the atoms C(3a) and C(6a) of the COT periphery by internal bonding. With our focus on the effect of this and similar internal covalent connectors on the frontier orbitals, we refer to the effect of the resulting orbital energy change as hybridization energy (*E*_hybr_). There should be no confusion with respect to the orbital mixing on the same atoms that is usually referred to as hybridization: in all molecules under discussion the atomic hybridization of the carbon atoms is sp^2^. *E*_hybr_ corresponds to the difference between the formation energy of the [4*n*]annulene reactant and of the hybridized product, an amount that is directly proportional to the HOMO–LUMO energy gap, *E*_H–L_. The *E*_H–L_ values, which are easily calculated at the Hückel level, are shown in Table 1 for all discussed compounds. Consequently, either *E*_hybr_ or *E*_H–L_ will give account of the effect of the connecting bonds relative to the antiaromatic [4*n*]annulene periphery. A trade‐off is established between *E*_hybr_>0 that acts against the formation of the open‐shell configuration and *E*_corr_<0 that stabilizes the non‐bonded electron pair diradicaloids.

**Table 1:** *E*_H–L_ defined as the *E*(HOMO)–*E*(LUMO) energy gap (related to *E*_hybr_) is evaluated at the Hückel level (in β units). *y*_0_ is computed by Equation (1).^[19]^ JT values (see Supporting Information) are obtained by using an alternating series of Hückel β values of 0.9 and 1.1 according to the bond alternation pattern of the respective [4*n*]annulene.^[a,b]^

	*E*_H–L_	*y*_0_		*E*_H–L_	*y*_0_
**1**	0	1	**11**	0.29	0.316
**2**	0.47	0.214	**12**	0.45	0.287
**2‐JT**	0.61	0.140	**13**	0.14	0.643
**3**	0.66	0.084	**14**	1.00	0.058
**4**	0.30	0.228	**15**	0	1
**5**	0	1	**16**	0.66	0.086
**6**	0.31	0.199	**17**	0.27	0.150
**7**	0.16	0.575	**18**	0.50	0.112
**8**	0.62	0.299	**19**	0	1
**8‐JT**	0.69	0.295	**20**	0.82	0.064
**9**	0.90	0.088	**21**	0.34	0.159
**10**	0.45	0.121	**22**	0.38	0.117

[a] For phenalenyl (**14**) and olympicene (**16**) *y*_0_ represents triradical character; for triangulene (**20**) *y*_0_ represents pentaradical character. For all other molecules, *y*_0_ represents diradical character. [b] For **14**, **16**, and **20** the HOMO to SOMO energy gap is shown.

The degree of open‐shell character can be described by the *y*_0_ index^[19]^ calculated as the natural orbital occupation number (NOON) of the LUNO (NOON_LUNO_, LUNO=lowest unoccupied natural orbital), as:

(1) $$y{}_{0}=\mathrm{NOON}{}_{\mathrm{LUNO}}$$

Note that *y*_0_=0 for closed‐shell molecules, and *y*_0_=1 for full diradicals, and 0<*y*_0_<1 for diradicaloids. The *y*_0_ values for all the systems discussed herein, which are shown in Table 1, are calculated at the same level of theory to provide feasible comparisons. This must be highlighted as the values of the *y*_0_ theoretical parameter strongly depend on the calculation method; thus, the *y*_0_ shown here can significantly differ from those reported in the literature for the same diradicaloid. *y*_0_ data have been obtained by ab initio electronic structure calculations (see Section 2 of the Supporting Information). Particular values of *E*_corr_ are not required in our discussion given that they are implicitly accounted for in the ab initio calculations to yield *y*_0_. We also address the role of the second‐order Jahn–Teller distortions (JT, also referred to as pseudo‐Jahn–Teller effect) that can contribute to stabilization through bond localization.^[20]^

Aside from the [4*n*]annulenes as the origins of the disjoint character, two other well‐known disjoint molecular fragments that produce diradicaloid structures are listed in Scheme 1: tetramethyleneethane (TME) and tetramethylenebenzene (TMB).[^2^, ^3^, ^4^] These also have degenerate disjoint frontier orbitals ready to develop a diradical character. Trimethylenemethane (TMM) and *meta*‐quinodimethane (mQDM)[^2^, ^3^, ^4^] also shown in Scheme 1 have non‐bonding degenerate frontier orbitals even though these are non‐disjoint.

Figure 1b illustrates two steps for *D*_2*h*_ pentalene (**2**) along the way as we introduce its diradical character. First, a non‐zero HOMO–LUMO energy gap, *E*_H–L_, arises due to the internal connecting bond. It is only because of the doubly excited configuration that a degree of diradical character remains; otherwise, the system would be closed shell. This H,H→L,L configurational mixing illustrated with the ⊕ symbol lends a degree of diradical character. In this case, as for the parent [8]annulene, the development of the diradical character, i.e., electron correlation, as resulting from the avoidance of the two electrons into separate regions is promoted by the reduced Coulomb interaction. Clearly, there is a balance between *E*_H–L_ and *E*_corr_ driving a certain degree of diradical character and *y*_0_ value.[^1^, ^21^] In other words, when *E*_H–L_ and *E*_corr_ are of comparable magnitude, the final electronic structure has significant diradicaloid character.

This situation is confirmed for *D*_2*h*_ pentalene by a value of *y*_0_=0.214 in correspondence with a *E*_H–L_=0.47 in Table 1. The development of the diradical character in **2** is further modulated by an additional energy reduction owing to the gaining of covalency (*E*_H–L_ in Figure 1c) by a second‐order Jahn–Teller effect resulting in the experimentally observed^[22]^ *C*_2*h*_ symmetry (**2‐JT**). This in turn: (i) strengthens the bonding coupling between the 1/2, 3/3a, 4/5 and 6/6a atom pairs in the HOMO essentially generating a bond length alternation, (ii) further increases the *E*_H–L_(*C*_2*h*_) gap to 0.61 in Table 1 for **2‐JT**, and (iii) consequently diminishes the disjoint property somewhat. All of this is nicely accounted for by a reduction of the diradical character to *y*_0_=0.140 for **2‐JT**.

From this description, some general trends can be derived. Starting from the zero‐gap [4*n*]annulene, and then (i) given the small inter‐orbital electron–electron repulsion between disjoint orbitals in the diradicaloid state, the singlet‐triplet gaps are also small; (ii) the changes of the [4*n*]annulene structure due to the internal covalent bonds by *E*_hybr_ are what make the optical HOMO→LUMO excitation appear at low energies (in the visible/near‐infrared region).^[23]^ In addition, (iii) this excitation is associated with weak or very weak absorption bands due to the disjoint character of the two relevant orbitals generating a small but not zero orbital overlap contribution to the electric dipole transition moment. Features (i), (ii), and (iii) are widely observed in this class of compounds and will be described further for diradical molecules reviewed herein.

**Figure 2:**
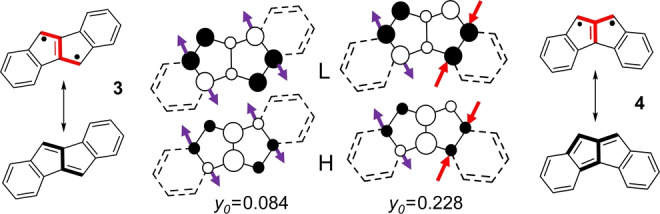
HOMO (H) and LUMO (L) orbitals of **3** and **4** constructed from **2‐JT**. Arrows indicate the atoms perturbed by the two benzene fusions. Pairs of purple/red arrows indicate destabilization/stabilization of the pentalene core orbitals. Valence bond (VB) structures are depicted with the substructures (i.e., TMM) highlighted in red.

Derivatives of pentalene obtained by lateral benzo‐annulation, illustrated in Figure 2,[^24^, ^25^, ^26^] can be mentioned. In **3**, benzo‐fusion onto the 2/3 and 5/6 bonds of pentalene, given their antibonding interaction in the *C*_2*h*_ structure, will destabilize the HOMO and LUMO^[27]^ orbitals (more the LUMO as its orbital coefficients are larger), enlarging the *E*_H–L_ gap (0.47→0.66 in Table 1) and decreasing *y*_0_ to 0.084. In **4**, the two benzenes are fused to the 2/3 and 4/5 bonds; for the latter bond, with bonding interactions in the HOMO and LUMO, fusion stabilizes them both, but the LUMO to a larger degree. This results in a reduction of the *E*_H–L_ gap (0.47→0.30 in Table 1) with a concomitant small increase of *y*_0_ to 0.228. Figure 2 displays the relevant valence bond structures, displaying a TMM fragment in **4** vs. a linear conjugated ethylene diradical in **3**, a difference contributing to the larger *y*_0_ of **4** vs. **3**.

Dinaphtho‐fused derivatives of pentalene have been implemented as semiconducting substrates in organic field‐effect transistors by Kawase and Takimiya.^[25]^ The best performing of these materials works as a unipolar p‐type semiconductor given their ease toward electrochemical oxidation.

## 3 Heptalene Diradicaloids

**Figure 3:**
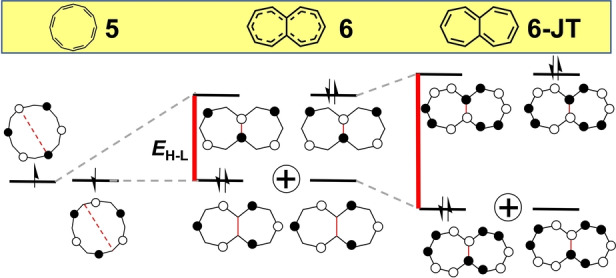
Evolution of the frontier molecular orbitals from planar [12]annulene, **5**, to planar *D*_2*h*_ heptalene, **6** and to planar *C*_2*h*_ **6** by second‐order Jahn–Teller distortion (**6‐JT**). Red lines denote *E*_H–L_. The H,H→L,L mixing is denoted as the sum of two configurations.

Compared to COT, the larger size of [12]annulene (**5**, Figure 3) permits a greater diversity of antiaromatic hybridized derivatives. At the planar *D*_12*h*_ geometry, this annulene displays degenerate SOMO orbitals with non‐bonding and disjoint characters (Figure 3), making it a Hückel diradical.

In analogy with COT and pentalene, heptalene^[28]^ (**6** in Figure 3) is the seven‐member bicyclic [12]annulene analogue with one single bond internally connecting the [12] π‐periphery. Upon hybridization of planar **5**, the degeneracy of its two SOMO orbitals is lifted giving rise to *D*_2*h*_ heptalene **6** characterized by a *y*_0_=0.199. In contrast to *D*_2*h*_ pentalene, where the two internally connected carbon atoms have the same orbital phases, in heptalene they have the opposite phases. The resulting *E*_H–L_=0.31 (Table 1 for *D*_2*h*_ heptalene **6**, see also the red lines in Figure 3) compares with *E*_H–L_=0.47 in pentalene **2**, giving way to a species with similar diradical characters: *y*_0_=0.199 for **6** vs*. y*_0_=0.214 for **2**. The bond‐equalized *D*_2*h*_ structure of **6** is unstable and undergoes second‐order Jahn–Teller distortion towards a *C*_2*h*_ bond localized form (**6‐JT**) illustrated in Figure 3 with the subsequent increase of *E*_H–L_ from 0.31 to 0.49 eV and a concomitant decrease of the diradical index from 0.199 to *y*_0_ *=*0.115.^[29]^ Heptalene displays a low energy absorption band in the NIR spectral region corresponding to a very weak feature again in line with its antiaromatic nature.^[30]^

**Scheme 2:**
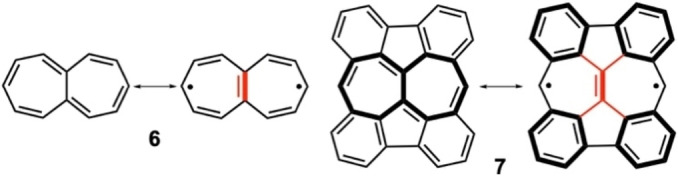
Planar heptalene (**6**) and its tetrabenzo derivative (**7**) and their diradical resonance forms. A synthesized derivative of molecule **7** has the hydrogens at the radical positions replaced by mesityl groups.^[31]^

Scheme 2 displays a tetrabenzo‐annulated derivative of heptalene, **7**. *E*_H–L_ upon benzene fusion onto the seven‐membered rings in positions with non‐bonding characteristics is reduced, *E*_H–L_=0.16 (Table 1). In the closed‐shell structure of **7**, however, two *ortho*‐quinoidal benzenes are formed which provide an additional pro‐aromatic driving force (see next section), increasing significantly the diradical character of **7** up to *y*_0_=0.575 in concordance with its smaller *E*_H–L_. The dimesityl derivative of **7** has been prepared (mesityls attached to the carbon atoms with high radical character),^[31]^ which shows an absorption band at 934 nm with very low absorbance.

## 4 s‐Indacene Diradicaloids: the Pro‐aromatic Driving Force

**Figure 4:**
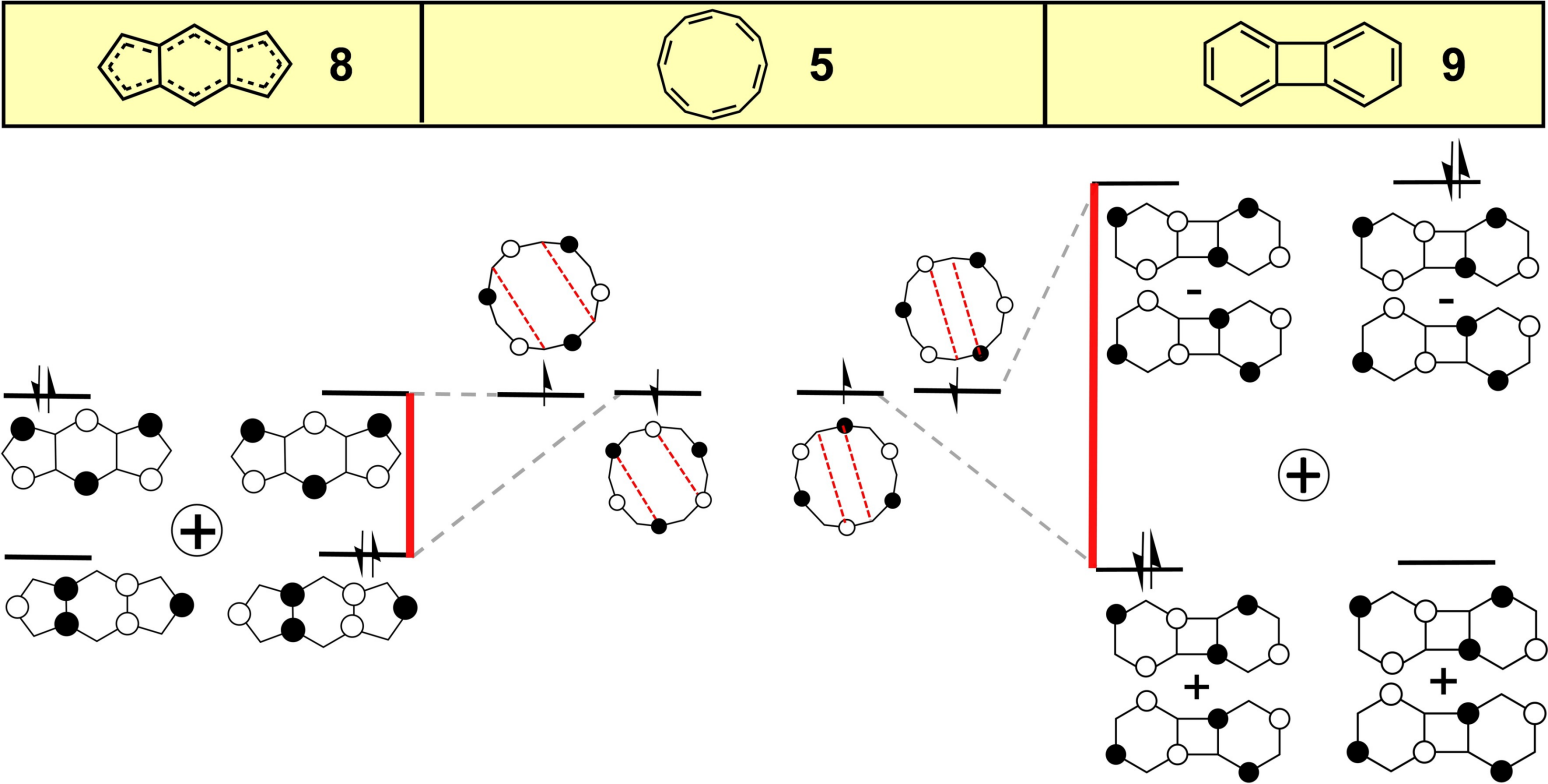
Evolution of the frontier molecular orbitals from planar [12]annulene (middle) to *s*‐indacene (**8**, *D*_2*h*_, left) and to biphenylene (**9**, right). *E*_hybr_ is shown in red. The H,H→L,L mixing is denoted as the sum of two configurations.

Inclusion of a benzenoid unit between the two five‐membered rings of pentalene produces tricyclic *s*‐indacene, **8**, shown in Figure 4.^[32]^ Like heptalene, *s*‐indacene **8** evolves from the antiaromatic [12]annulene periphery with a mode of annulene hybridization that involves two connecting bonds, leading to significant differences.

**Figure 5:**
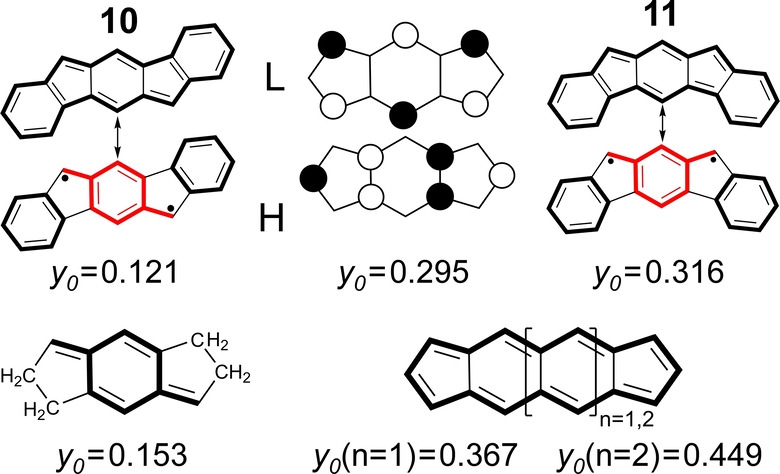
Top: frontier orbitals of *C*_2*h*_ s‐indacene and of indenofluorenes **10** and **11**. Bottom: *para*‐QDM model of *s*‐indacene (left) together with the naphtho‐ (*n*=1) and anthra‐ (*n*=2) analogues of *s*‐indacene (*n*=0).

The formation of two Ca−Ca′ and Cb−Cb′ bonds (Scheme 1) in *s*‐indacene opens a *E*_H–L_ gap between the disjoint orbitals that is larger than in pentalene (0.47 vs. 0.62 in Table 1). This is attributed to the two bonding interactions by connecting atoms with the same phases (i.e., HOMO) while the other orbital (i.e., LUMO) remains unaffected, as shown in Figure 4. The *D*_2*h*_→*C*_2*h*_ second‐order Jahn–Teller distortion in *s*‐indacene (**8‐JT** in Table 1) does not affect *y*_0_ much: the values are 0.299 (*D*_2*h*_) vs. 0.295 (*C*_2*h*_). A new aspect in *s*‐indacene is the formation of a central six‐membered ring contributing with a benzoquinoidal unit, which opens another source of diradicaloid character by aromatic stabilization (Figure 5). This would justify that *D*_2*h*_ *s*‐indacene displays a diradical character, *y*_0_=0.299, which is larger than that of pentalene despite the larger *E*_H–L_ gap in the former. The aromatic diradical character is manifested by the non‐negligible *y*_0_=0.153 value in a model compound of *s*‐indacene having only a quinodimethane π‐unit (Figure 5). In this regard, going from *s*‐indacene to the naphtho‐ (*n*=1, *y*_0_=0.367)^[33]^ and to the anthra‐quinodimethane (*n*=2, *y*_0_ *=*0.449) derivatives, shown in Figure 5, *y*_0_ increases in parallel with the increase of aromatic stabilization in the central acene. For a similar discussion of the orbital structure of *as*‐indacene, the diradicaloid isomer of *s*‐indacene, see Section 3 of the Supporting Information.

Lateral dibenzo‐fusion of *s*‐indacene in Figure 5 gives rise first to indenofluorene **10**,^[34]^ where *y*_0_=0.121 is smaller than *y*_0_=0.299 in *s*‐indacene, an evolution similar to that of pentalene (**2‐JT**)→**3**. Given that dibenzo‐annulation in **10** does not substantially affect the central diradical aromatic unit, the reduction of *E*_H–L_ in Table 1 (0.45) compared to *s*‐indacene (0.62) is not accompanied by an increase of *y*_0_, but rather by a decrease. To account for this, it can be argued that the increment of the total number of peripheral π‐electrons, from 4*n*=12 to 4*n*=20, decreases *E*_corr_ given the reduced correlation effect since the Coulomb repulsion is reduced due to the normalization of the orbital atomic coefficients. Changing the dibenzo‐annulation pattern from **10** to **11**[^22^, ^35^] generates a value of *y*_0_=0.316 that is consistent with the decrease of *E*_H–L_ from 0.45 to 0.29. In **11**, a *m*QDM diradical substructure is generated (Figure 5), versus a central *p*QDM unit in **10**. *m*QDM is listed in Scheme 1 as one of the common promoters of diradical character.

Biphenylene,^[36]^ **9** in Figure 4, is also obtained by forming two C−C bonds between two pairs of consecutive central atoms of [12]annulene. This connection between non‐bonding atomic orbitals of the two degenerate SOMOs of **5** causes the resulting two molecular orbitals to mix further and their symmetric and antisymmetric linear combinations give rise to the two frontier molecular orbitals of **9** (Figure 4). The resulting *E*_H–L_=0.90 for biphenylene is larger than that of heptalene and of *s*‐indacene. For this reason, its diradical character, *y*_0_=0.088, is much smaller than those of its congeners. Another consequence of the larger *E*_H–L_ for biphenylene is that its electronic absorption spectrum has its lowest energy band at much higher energy than its parents, yet it is still very weak revealing its antiaromatic origin.^[37]^ Comparing the three [12]annulene derivatives, we find that the *y*_0_ of biphenylene (*y*_0_=0.088) is smaller than that of heptalene **7** (*y*_0_=0.199) due to the larger *E*_hybr_ which is still smaller than that of *s*‐indacene **8** (*y*_0_ *=*0.295) due to the pro‐aromatic contribution in the latter.

**Scheme 3:**
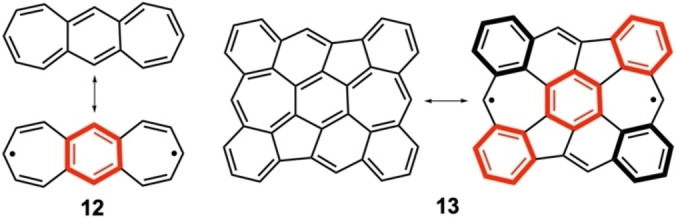
VB structures of derivatives **12** and **13** of heptalene.

Compound **13**[^38^, ^39^] (Scheme 3) is the tetrabenzo‐fused variant of **12** ^[40]^ (**12** is the heptalene analogue of *s*‐indacene). The diradical character of **12** amounts to *y*_0_=0.287, which is larger than that of heptalene due to the aromatic contribution in the center (in **12**, *E*_H–L_ **=**0.45 and in **6**, *E*_H–L_ **=**0.31). At the same time, the diradical character of **13**, *y*_0_=0.643, is even larger than that of **12** because of the added aromaticity of the additional benzenes in the periphery, leading to a very small *E*_H–L_=0.14. **13** has been recently prepared on Au(111) surface and characterized by scanning electron microscopy as an open‐shell species.^[39]^

Further derivatives of biphenylene and of extended *s*‐indacenes have been successfully used as semiconductor substrates in organic field‐effect transistors (OFETs), mostly displaying unipolar electrical p‐type conduction.[^25^, ^41^, ^42^] In this regard, a diradicaloid diindenoanthracene analogue (i.e., dimesityl bis(triisopropylsilyl)ethynyl dibenzo derivative of the *n*=2 system in Figure 5) has been shown to work as an ambipolar semiconductor exhibiting p‐ and n‐conduction.^[43]^ This electrical behavior is connected to reversible oxidations and reductions seen in the cyclic voltammetry. Diradicaloids with medium diradical characters can stabilize either holes or electrons in a similar way because of the substantial reduction of the intramolecular reorganization energies compared to their closed‐shell forms. Redox amphoterism and reduced reorganization energies are key ingredients for ambipolar behavior, properties that are uniquely provided by diradicaloids.^[44]^

## 5 Benzenoid Polycyclic Compounds from [4n]Annulenes: Phenalenyl and Olympicene

**Figure 6:**
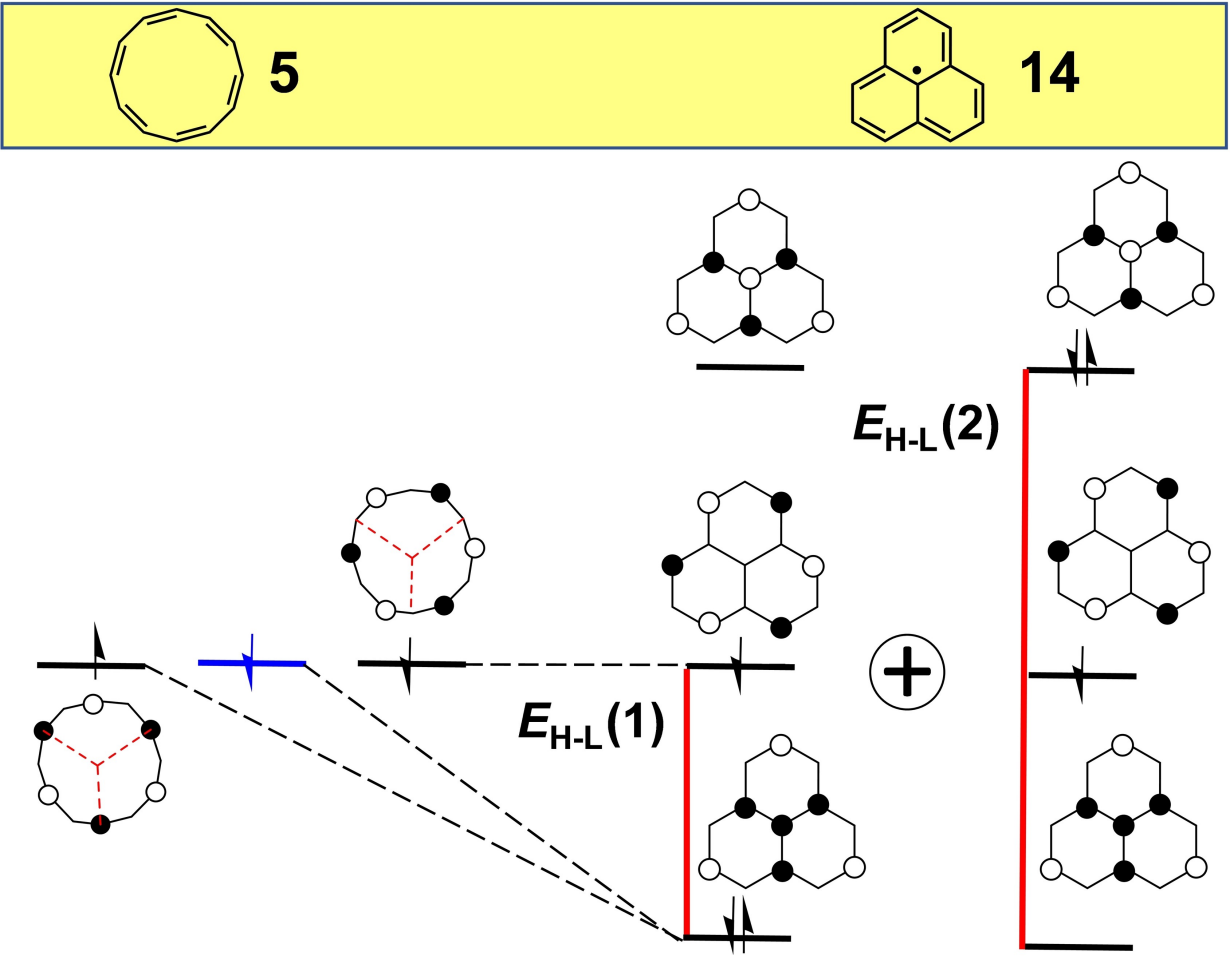
Evolution of the frontier molecular orbitals from planar [12]annulene to phenalenyl radical, **14**. The non‐bonding orbital of a single π ‐atomic orbital is in blue. Two different *E*_H–L_ are shown. The H,H→L,L mixing is denoted as the sum of two configurations.

The phenalenyl radical, **14** in Figure 6, is a tricyclic benzenoid compound composed of a total number of 13 electrons that can be viewed as a [12]annulene periphery plus one internal carbon atom.^[45]^ The [4*n*]annulene origin of **14** is revealed in the orbital pattern of its SOMO orbital that has the same pattern as that of [12]annulene (Figure 6). The other SOMO orbital of [12]annulene evolves in phenalenyl by bonding coupling with the central carbon atom orbital that produces a stabilization as illustrated by *E*_H–L_(**1**) in Figure 6. This HOMO orbital in phenalenyl is one of the nearly triply degenerate set of doubly‐occupied orbitals (only one of the three is shown) below its SOMO.

Since the starting point in phenalenyl is a radicaloid structure, the balance between *E*_corr_ and *E*_hybr_ would give rise to triradical character occurring by the H,H→L,L mixing between the doubly occupied and the empty orbitals through the *E*_H–L_(**2**) energy gap in Figure 6 (only E_H–L_(**1**)=1.00 is listed in Table 1, *E*_H–L_(**2**)=2.00). The large *E*_H–L_(**2**) makes the triradical character very small (LUNO occupation number is NOON_LUNO_=0.058) despite of the fact that the HOMO and LUMO orbitals of phenalenyl radical have disjoint and non‐bonding characters along the annulene periphery.

The large hybridization between the central π ‐atomic orbital and the annulene periphery in **14** results from the three in‐phase orbital interactions. In this regard, the phenalenyl radical is our first benzene‐based system with an antiaromatic periphery. Phenalenyl radical is a famous building block in organic electronics for which pioneering studies of magnetism in organic materials were carried out.[^46^, ^47^, ^48^]

**Figure 7:**
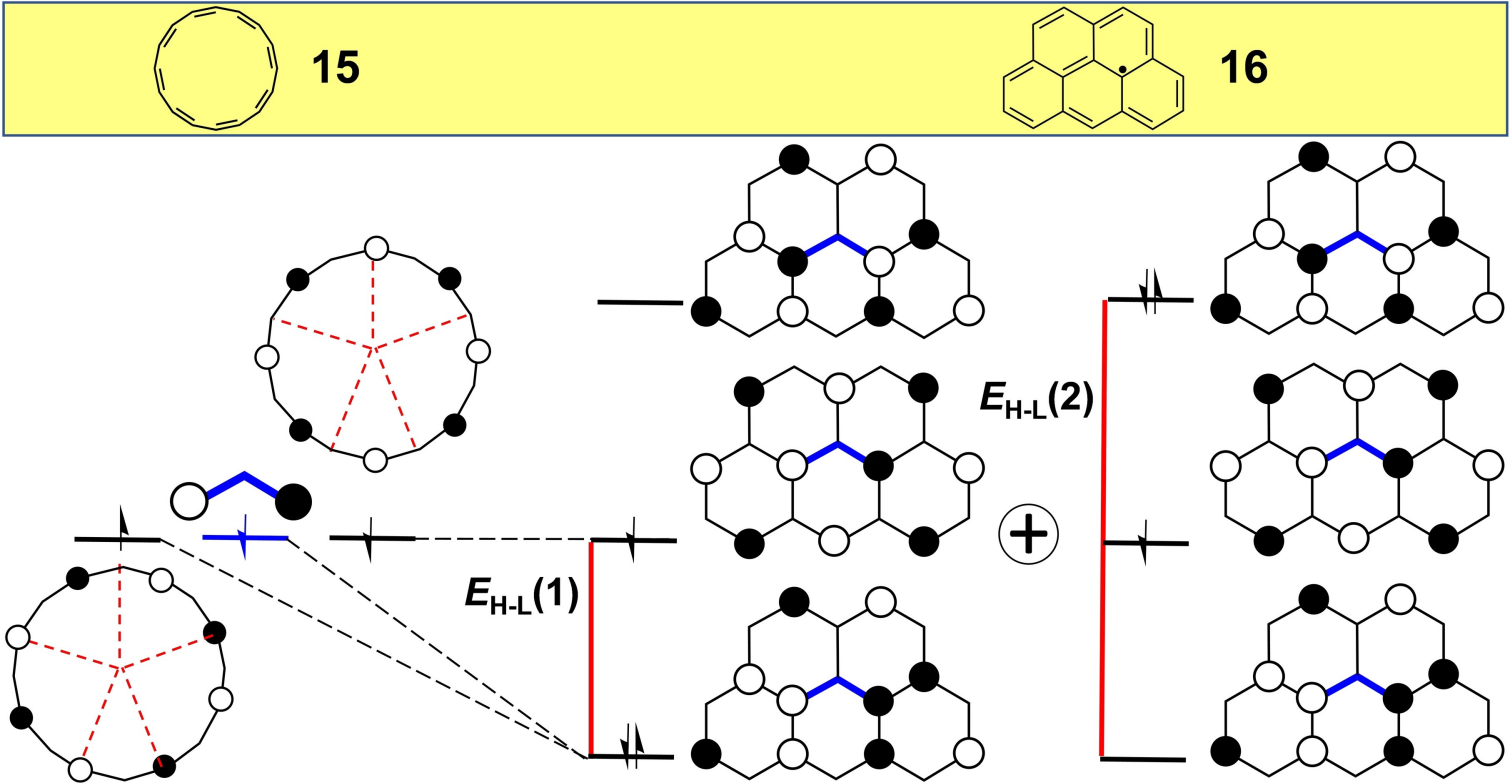
Evolution of the frontier molecular orbitals from planar [16]annulene, **15** to olympicene, **16**. Two different *E*_H–L_ gaps are shown. The H,H→L,L mixing is denoted as the sum of two configurations.

Recent developments, first in surface chemistry and then in solution organic chemistry, have made it possible to prepare derivatives of olympicene, compound **16** in Figure 7.^[49]^ Frontier molecular orbitals of olympicene emerge from hybridization of the SOMO orbitals of [16]annulene, **15**, with the SOMO orbital of the allyl radical (blue in Figure 7). Bonding interaction between the orbitals of the periphery and of the central fragment produces an *E*_H–L_=0.66. In addition, the SOMO of olympicene emerges by the combination of the allyl moiety through the non‐bonding atoms of the periphery generating an orbital with similar energy to that of the SOMO of the starting [16]annulene. The LUNO occupation number of olympicene is NOON_LUNO_=0.086 in agreement with an almost pure radicaloid character.

## 6 Complex π‐Forms Made from [4n]Annulene Peripheries

The concept developed from the primordial antiaromatic [4*n*]annulene structure through a diversity of “covalent” hybridizations (i.e., by forming one, two and three σ‐bonds) can be recognized in an assortment of complex molecular systems.

**Scheme 4:**
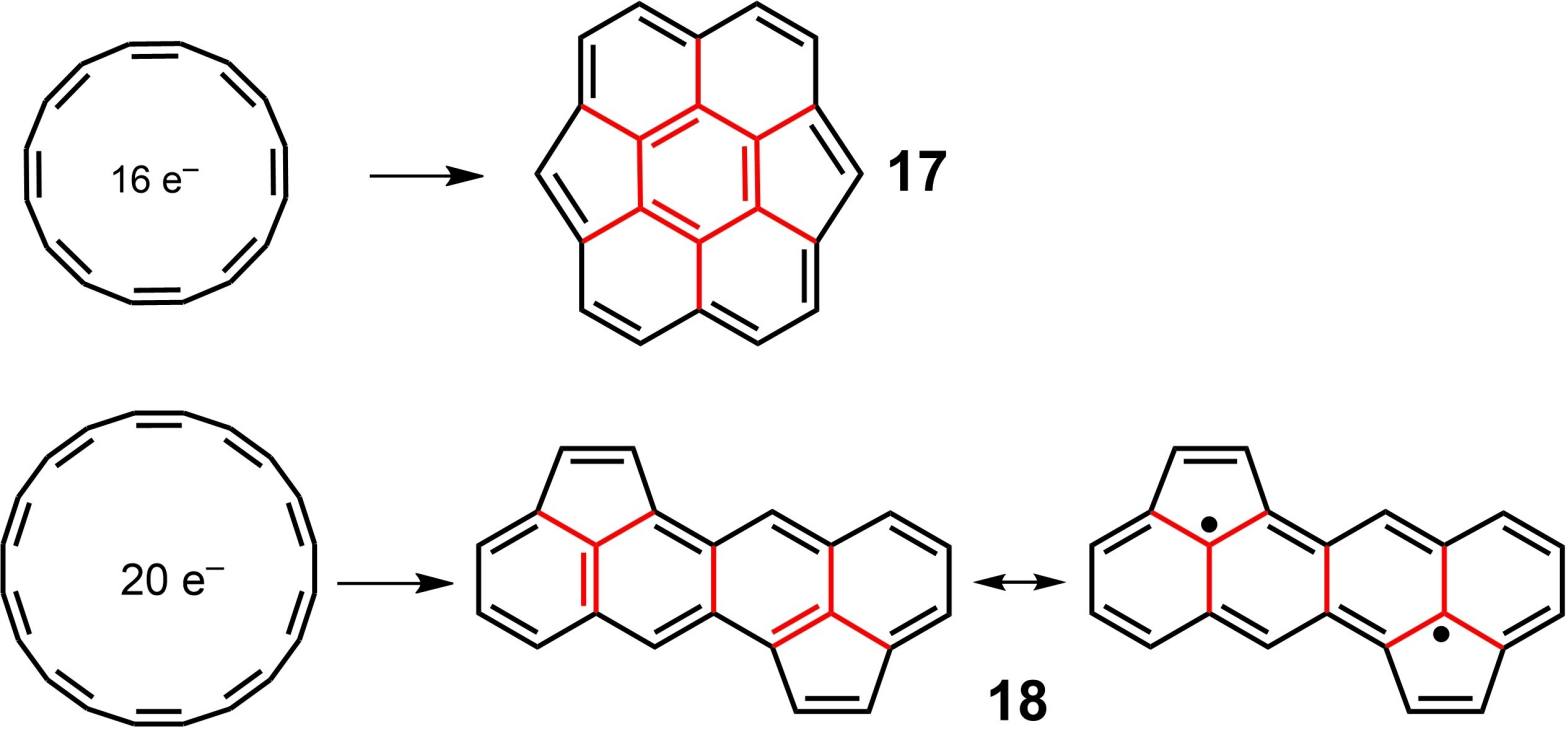
Hybridization of [16]annulene and [20]annulene with covalent connecting bonds (in red) leading to unusual polycyclic hydrocarbons **17** and **18**, respectively.

By adding suitable internal covalent connections, one can generate, for example, molecules **17** and **18** (Scheme 4) from [16]annulene and [20]annulene, respectively. Derivatives of both **17** ^[50]^ and **18** ^[51]^ have been recently prepared. Both molecules display electronic absorption spectra with bands at very low energy at 770 nm (with a long absorption tail up to 1000 nm) in **17** and at 680 nm (with tail up to 830 nm) in **18**, which are characteristically weak and broad following the spectral profile pattern expected for antiaromatic non‐alternant molecules. Pentacene homologues of **18** with peripheral 4*n*=20 have also been prepared by the Plunkett group.^[52]^ The presence of multiple internal bonds in **17** and **18** (red lines in Scheme 4) promote hybridization energies amounting to *E*_H–L_=0.27 and 0.50, respectively, resulting in small diradical characters (*y*_0_=0.150 for **17** and *y*_0_=0.112 for **18**, respectively). The diradical resonance structure for **18** is shown in Scheme 4.

**Figure 8:**
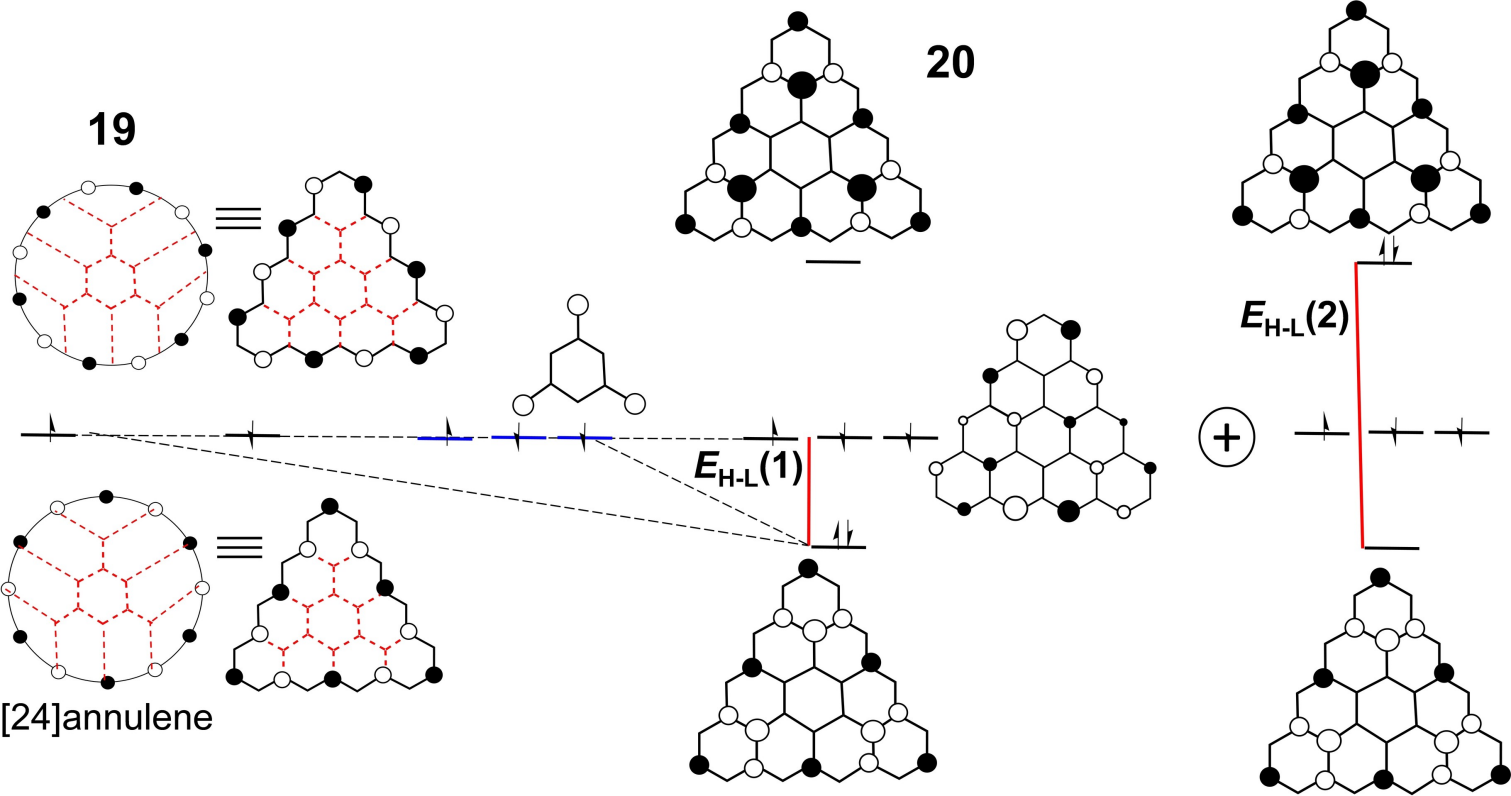
Evolution of the frontier molecular orbitals from planar [24]annulene (**19**) to [4]triangulene (**20**). Two different *E*_H–L_ gaps are shown. The H,H→L,L mixing is denoted as the sum of two configurations.

**Figure 9:**
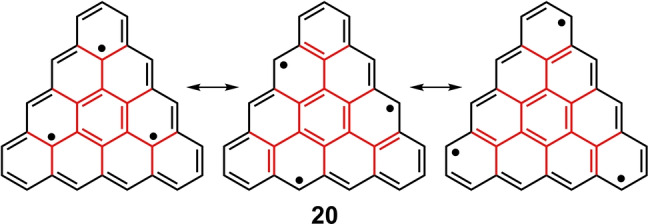
Selected resonance forms of **20** highlighting the placement of the three unpaired electrons either in the annulene periphery and in the inner core.

Starting from the phenalenyl radical, the next hierarchically higher members of the triangular‐shaped series of compounds, the so‐called [*n*]triangulenes (*n* is the number of benzenes in one of the sides of the triangle), can be constructed. Some of these [*n*]triangulenes possess a 4*n* π‐electron periphery, the one in our discussion is [4]triangulene **20** (Figure 8), derived from the periphery of [24]annulene, **19**. Molecule **20** has been recently prepared by surface synthesis and has its 24 peripheral π‐electrons internally hybridized by a 1,3,5‐trimethylenebenzene motif.^[53]^ The construction of the relevant frontier orbitals of **20** starts from the two SOMO orbitals of [24]annulene, similar to the previously discussed cases above, to accommodate one electron each at the Hückel level, making the ground state a triradical species with three nearly degenerate orbitals.^[54]^ The strong hybridization, as in the other triangulenes, leads to a large *E*_H–L_=0.82. The resulting small H,H→L,L mixing is then revealed by the low value of NOON_LUNO_=0.064. Selected canonical resonance forms carrying the triradical character are shown in Figure 9.

## 7 From Non‐alternant Disjoint Diradicals to Edge‐segregated Disjoint Diradicals

**Figure 10:**
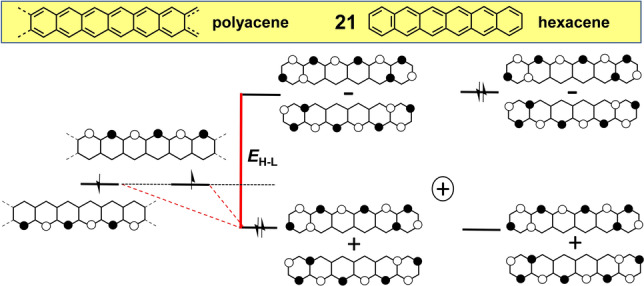
From polyacene to hexacene **21**. Here, the evolution process giving rise to hybridization of the frontier orbitals consists of “fragmenting” the polyacene structure into terminal and middle groups.

Hexacene (**21**) with 26 π‐electrons (Figure 10) can be taken as a representative example of the family of acenes[^55^, ^56^] formed by fusion of benzene rings with a [4*n*+2]annulene periphery.

Acenes share some similarities with [4*n*]annulenes in the sense that these molecules partially feature non‐bonding and disjoint patterns in their frontier orbitals along the longitudinal molecular zig‐zag edges where the density of the unpaired electrons is also localized.^[57]^ This orbital structure is highlighted in the extreme case of polyacene,^[58]^ the infinitely long acene chain showing the two degenerate non‐bonding orbitals (NBOs) displayed on the left in Figure 10. In oligoacenes, terminal rings break the non‐bonding/disjoint character of the edge orbitals imparting a hybridization effect (*E*_H–L_=0.34 for **21**) that promotes electron pairing but is diminishing with increasing size of the acene homologues. Hence, the electronic structure of oligoacenes is a hybrid between an open‐shell configuration favored by the non‐bonding edge orbitals and the closed‐shell structure fuelled by terminal hybridization. The resulting compromise is that the frontier orbitals in oligoacenes keep the disjoint and non‐bonding characters along the edges, an effect that increases with the size of the oligoacene, which is referred to as edge orbital segregation.^[59]^ Both non‐bonding and disjoint features make long acenes open‐shell diradicals (*y*_0_=0.159 in **21**) with an increasing multiradical character with increasing size.^[57]^

**Figure 11:**
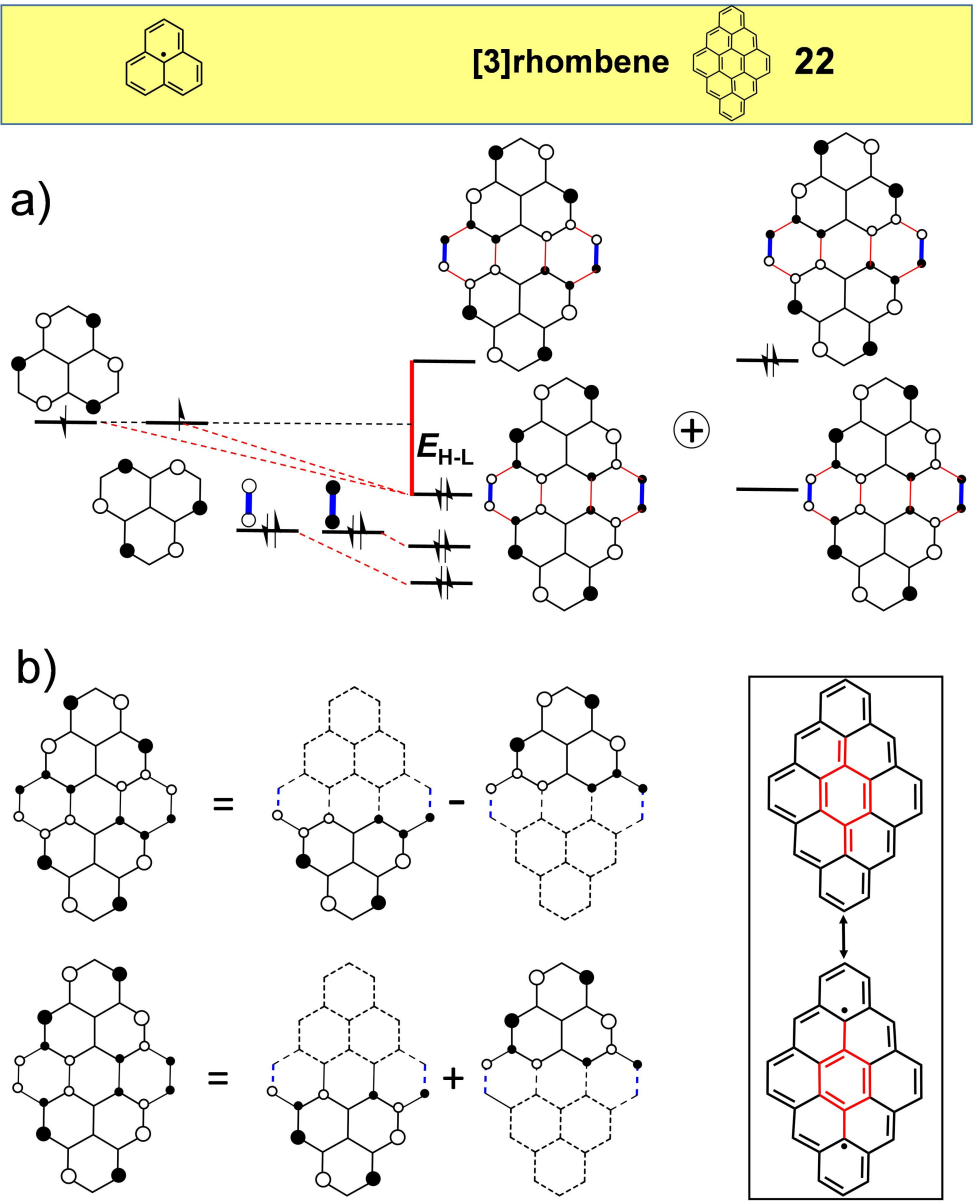
a) from phenalenes to [3]rhombene, **22**, with its relevant frontier orbitals and valence bond structures. b) left: construction of the Hückel HOMO (bottom) and LUMO (top) orbitals of **22** from the symmetric and antisymmetric combinations of phenalenyl orbitals, respectively. right: resonance forms of [3]rhombene.

Similar to hexacene, compound **22**, from the family of [*n*]rhombenes (Figure 11), can be considered as a derivative of [22]annulene.^[60]^ The frontier orbitals of **22** can be derived by connecting two phenalenyl radicals through two ethylenes (shown in blue in Figure 11, top). Interestingly, the non‐bonding and disjoint property of the phenalenyl radical is to some extent preserved in the frontier orbitals of **22**. Indeed, its HOMO and LUMO orbitals are formed from the symmetric and antisymmetric combinations of phenalenyl‐like segregated fragments. Nonetheless, due to the [4*n*+2] π‐electron count, the HOMO and LUMO orbitals have fractions of bonding interactions in the annulene circuit co‐existing with the phenalenyl‐like non‐bonding orbital pattern. **22** contains a *para*‐quinodimethane unit hybridizing the whole periphery which further splits the energies of the frontier orbitals, overall resulting in an *E*_H–L_=0.38. The modest diradical character for **22** (*y*_0_=0.117 in Table 1) comes from two sources: on the one hand, the partial non‐bonding/disjoint orbital property that increases the H,H→L,L mixing and, on the other hand, the aromatic stabilization of the diradical structure in the *para*‐quinodimethane central unit (bottom right in Figure 11). [*n*]Rhombenes of different sizes have been recently prepared by surface synthesis.^[60]^

## 8 Conclusions

Herein, we highlight the concepts (i.e., disjoint and non‐bonding orbital characters, correlation effects and pro‐aromatic character) that connect the electronic structures and the sizeable diradical character in several families based on π‐extended polycyclic [4*n*]annulenes (which would be Hückel antiaromatics in their planar geometries). We explore the concept of hybridization of [4*n*] antiaromatic peripheries, connecting them with internal covalent bonds directly between atoms on the peripheries or with atoms or fragments. This orbital hybridization leads to HOMO–LUMO energy gaps that can be modest due to the particular disjointed property of the degenerate non‐bonding orbitals of the [4*n*] periphery. At the same time, the spatial separation of the two electrons can reduce their Coulomb repulsion stabilizing the diradicaloid character through a mixing with a doubly excited configuration, denoted as (H,H→L,L). This mechanism can be particularly effective for generating non‐alternant loops (as in **2**, **3**, **4**, **6**, **7**, **8**, **10**, **11**, **12**, **13**, **17**, **18**) helping to recognize interesting spectroscopic properties and useful applications. Other systems such as porphyrinoids,^[61]^ (norcorroles) and porphyrin‐based nanorings^[62]^ as well as circulenes[^63^, ^64^] also contain antiaromatic circuits; however, the degree of internal covalent bonding exceeds the limits of our perturbative approach.

On the one hand, we hope the present analysis will help the general reader to understand the origins of and to widen the range of the diradicaloid characters in new conjugated molecules that are now in rapid emergence given their technological applications in organic electronics. On the other hand, we highlight simple molecular design principles able to help produce a diversiform of complex and fascinating molecules that enlighten the immense beauty of Chemistry.

## Conflict of interest

The authors declare no conflict of interest.

## Biographical Information

*Sergio Moles Quintero was born in Nerja, Málaga (Spain) in 1991. He studied at the University of Málaga where he obtained the degree in Chemistry in 2016. Currently, he is on the last year of his Ph.D studies under the supervision of Prof. Juan Casado and Dr. José L. Zafra. His research is focused on the analysis of the electronic and vibrational structure of* π *‐conjugated Kekulé‐type diradicals*.

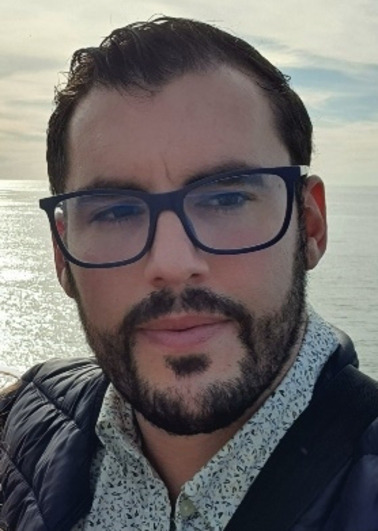

## Biographical Information

*Michael M. Haley received his B.A. (1987) and Ph.D. (1991) degrees from Rice University in Houston, Texas. After a postdoctoral stay at University of California, Berkeley, he joined the faculty at the University of Oregon in 1993 where he currently is the Richard M. & Patricia H. Noyes Professor of Chemistry. A co‐author of over 230 articles, Haley was recognized for his innovative hydrocarbon research with the 2021 ACS George A. Olah Award in Hydrocarbon or Petroleum Chemistry. His current research interests focus on the synthesis and properties of antiaromatic organic semiconductors and on phenylacetylene‐based molecular scaffolds for anion sensing*.

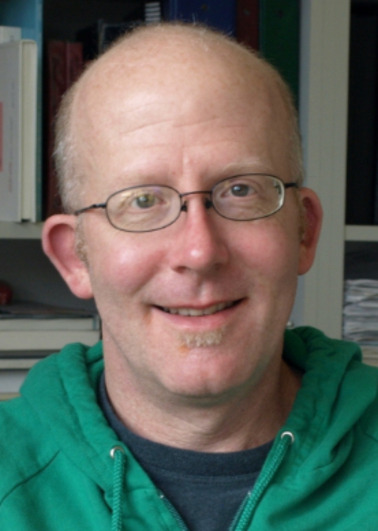

## Biographical Information

*Born in Budapest, Hungary, Miklos Kertesz studied at the Eötvös Loránd University, and joined the research staff of the Central Research Institute for Chemistry of the Hungarian Academy of Sciences. After postdoctoral studies in the US, he joined the faculty of the Chemistry Department at Georgetown University in 1982. His work focuses on understanding the structural, electronic, vibrational, and magnetic properties of molecules, polymers, and crystals. His interests include designing new materials with desirable physical properties, conducting organics, nanotubes, aromaticity, and intermolecular interactions in organic solids*.

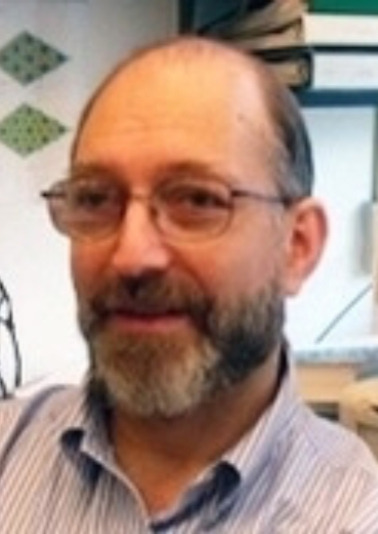

## Biographical Information

*Born in Antequera, Spain, Juan Casado studied Chemistry at the University of Málaga (UMA) where got the PhD in Chemistry with spectroscopic studies of oligothiophenes. After postdoctoral studies at University of Minnesota and at Steacie Institute for Molecular Sciences (NRC Canada in Ottawa), he joined the Department of Physical Chemistry at UMA where he is now Professor since 2016. His work focuses on understanding the electronic, vibrational, and magnetic properties of π*‐*conjugated molecules made of aromatic and antiaromatic units. In the last 10 years, his interests also include the understanding of the chiroptical properties of organic molecules*.

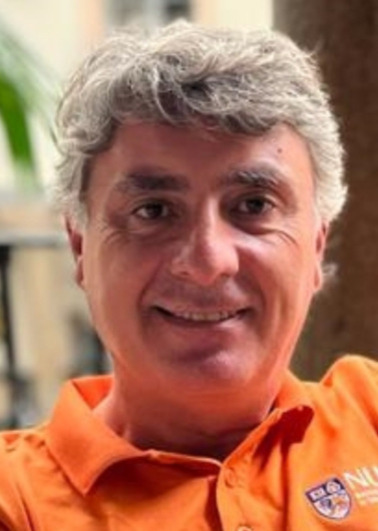

## Supporting information

As a service to our authors and readers, this journal provides supporting information supplied by the authors. Such materials are peer reviewed and may be re‐organized for online delivery, but are not copy‐edited or typeset. Technical support issues arising from supporting information (other than missing files) should be addressed to the authors.

Supporting Information
