# Supplementary material for: Polycyclic Hydrocarbons from [4n]Annulenes: Correlation versus Hybridization Forces in the Formation of Diradicaloids
Source: Angew Chem Int Ed Engl. 2022 Sep 14;61(44):e202209138. doi: 10.1002/anie.202209138 (PMC9826091; doi:10.1002/anie.202209138)
Supplement: Supplementary file 1 — Supporting Information [file ANIE-61-0-s001.pdf]

Angewandte Chemie
Eine Zeitschrift der Gesellschaft Deutscher Chemiker

Supporting Information

Polycyclic Hydrocarbons from [4*n*]Annulenes: Correlation versus Hybridization Forces in the Formation of Diradicaloids

S. Moles Quintero, M. M. Haley, M. Kertesz, J. Casado**

Supporting Information

Table of Contents Page

1. Correlation Energy S2
2. Computational Methods S4
3. *as*-Indacene S5
4. References S6
5. Cartesian Coordinates of Optimized Geometries S7

1. Correlation Energy

We compare two electronic configurations illustrated in Figure S1 within the spin-unrestricted formalism. Case I corresponds to the diradicaloid ground state, and Case II to the doubly occupied state. Assuming that the two SOMOs have the same integral (h) over the one-electron part of the Hamiltonian, the differences occur only in the electron-electron replulsion (Coulomb) part. J_{ij} refers to the Coulomb integral.

- **Case I** (DODS: different orbitals for different spins):

Ψ_{DODS}(1,2) =N det$|a\alpha b\beta|$, where $a(1) = \chi_1(1)$, and $b(1) = \chi_2(1)$. The π-molecular orbital χ_1 can be expressed in term of the π-type atomic orbitals, one on each carbon atom:

$$\chi_1 = \sum_{i=1}^{8} c_i \varphi_i$$

For χ_1 and χ_2 the values of $|c_i|$ =0 or 0.5 as illustrated in Figure S1. The energy contribution[1] is:

E_I=2h+ $<\chi_1\chi_2|\chi_1\chi_2>$=2h+ J_{12} ,

where electron-electron repulsion integral is $<\mathrm{xy}|\mathrm{zw}> = \iint x(1)\,y(2)\,\frac{1}{|r_1-r_2|}z(1)w(2)dV_1dV_2$.

- **Case II** (double occupancy, a=b):

Ψ(1,2) =N det$|a\alpha a\beta|$, where $a(1) = b(1) = (\chi_1(1) + \chi_2(1))/\sqrt{2}$.

E_{II}=2h+1/2$<\chi_1\chi_1|\chi_1\chi_1>$+1/2$<\chi_1\chi_2|\chi_1\chi_2>$= $2\mathrm{h} + 1/2\ (J_{11} + J_{12})$.

In the main text we use symbols to emphasize the separation of the electrons in the diradical configuration E_I =*E*(●/●), vs E_{II} = *E*(●●) for the closed shell configuraton.

The difference of the energy between the two configurations is

$$\Delta E = E_I - E_{II} = E_{\mathbf{corr}}(\text{disjoint}) = E(\bullet/\bullet) - E(\bullet\bullet) = -1/2(J_{11} - J_{12}).$$

Generally, $J_{11} - J_{12} > 0$, thus the diradicaloid state (E_I) is more stable than the closed shell state (E_{II}).

Some numbers can be generated by qualitatively starting from the Pariser-Parr-Pople Hamiltonian and looking at the Coulomb integrals. The exhaustive analysis of Shulten et al.[2] for example, uses for the on-site Coulomb integral, γ_{00}, a generally accepted value of 11.1 eV. For the first neighbor Coulomb integral, γ_{01}, they provide a range of values between 1 and 10 eV, with the more commonly used Ohno value being close to 10 eV, and the Mataga-Nishimoto approximation with the value being close to 6 eV.[2] Applying this approach for octatetraene, the following is obtained, further assuming that only first neighbor Coulomb integrals are retained:

$$J_{11} = 1/2(\gamma_{00} + \gamma_{01}),$$

$$J_{12} = \gamma_{01}/2$$

Applying the Mataga-Nishimoto values of $\gamma_{00} = 11\ eV$ and $\gamma_{01} = 6\ eV$

$$E_{corr}(\text{disjoint}) = E(\bullet/\bullet) - E(\bullet\bullet) = \Delta E = -2.75\ eV$$

indicating a significant energy advantage to the diradicaloid electronic configuration over the closed shell configuration for COT.

Figure S1. Electron configurations considered for the two SOMOs in cyclooctatetraene (D_{8h}) COT.

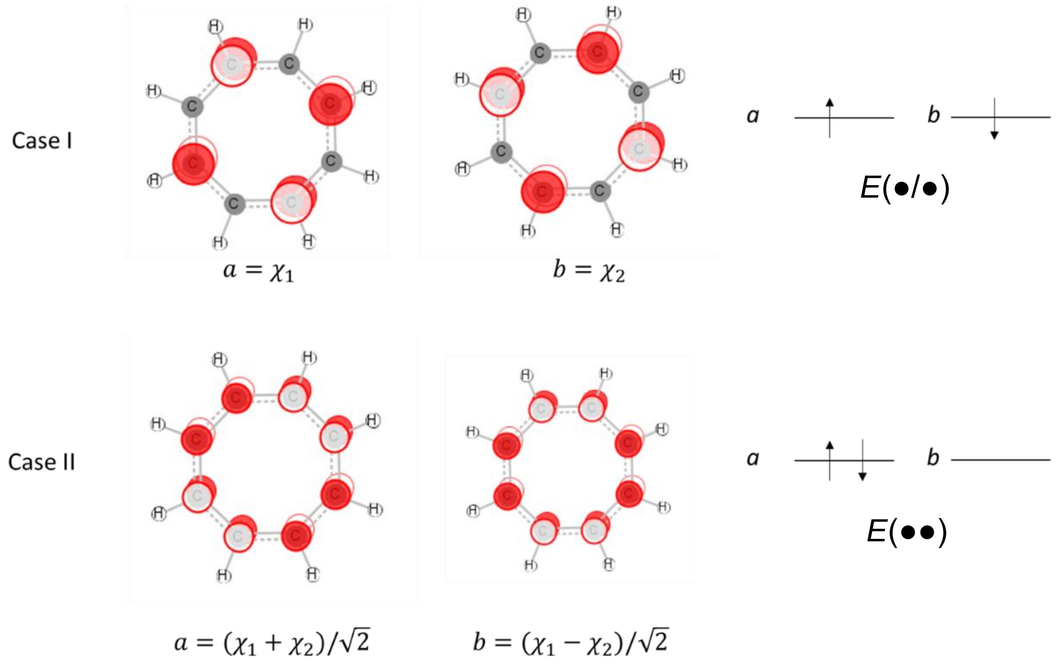

2. Computational Methods

Electronic structure calculations at the restricted active space spin-flip (RAS-SF) level of theory with 4 electrons in 4 orbitals in the active space and with the 6-31G(d) basis set were carried out. In the first step, the optimized geometry was calculated by DFT methodology at the (U)B3LYP/6-31G(d) level of theory. Then RAS-SF ab initio calculations were done with the Q-Chem program in the previously calculated DFT optimized geometry.[3] For the cases where the Jahn-Teller distortions were considered, the JT distorted geometry was calculated by restricting the geometry optimization calculations to the required geometry for the JT distortion. All frequencies were real except for the following, in agreement with the fact that these molecules are subject to JT distortions. The following is a list of imaginary frequencies:

2: *i* 1992 cm^{-1},
6: *i* 63 cm^{-1},
6JT: *i* 1756 cm^{-1} (note that heptalene further distorts out of the plane in the real JT distortion),
8: *i* 335 cm^{-1}.

A variety of parameters describing the diradical character of a molecule are available in the literature. While none of these parameters are physical observables,[4] they are useful and widely applied for comparative purposes. y_0 is one of these parameters to compare the diradical character of similar molecules. There are several ways to calculate y_0. In this work the Doehnert-Koutecký definition[5] has been used:

$$y_0 = N_{LUMO},$$

where N_{LUMO} is the lowest unoccupied natural orbital occupation number.[5,6] Hückel calculations were done by HuLis.[7] The bond length alternating Jahn-Teller distorted structures were referred to in Table 1 with the -JT suffix and were modeled in the Hückel calculations by β values of 1.1 and 0.9, except for the connecting bonds as defined in the main text which remain at β=1.0.

3. *as*-Indacene

We provide another application of our analysis for *as*-indacene.

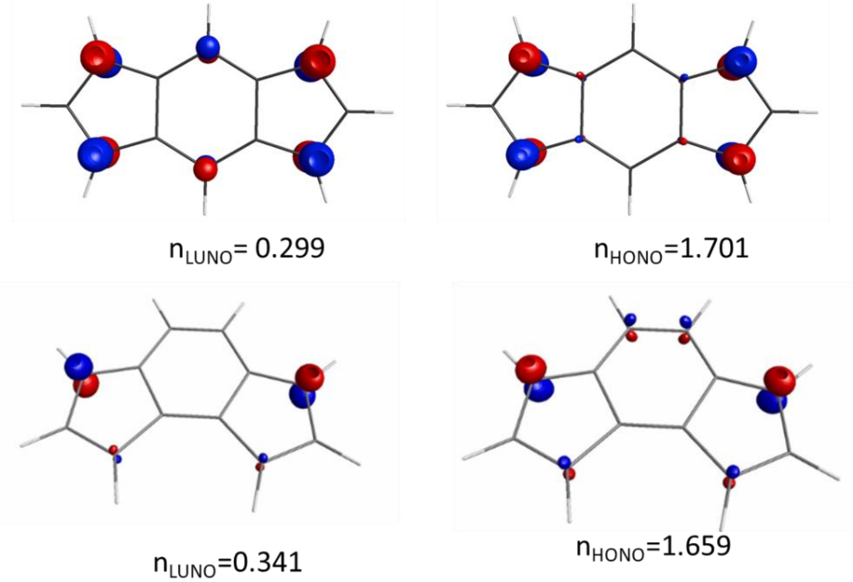

Figure S2. Natural orbitals and their corresponding occupation numbers for *s*-indacene (top row) and *as*-indacene (bottom row). Accordingly, $y_0 = 0.299$ for s-indacene and $y_0 = 0.341$ for s-indacene. Note that the Hückel gap for as-indacene is only 0.06 β and that the HOMO and LUMO are only partly disjointed. Molden[8] is used to represent the natural orbitals.

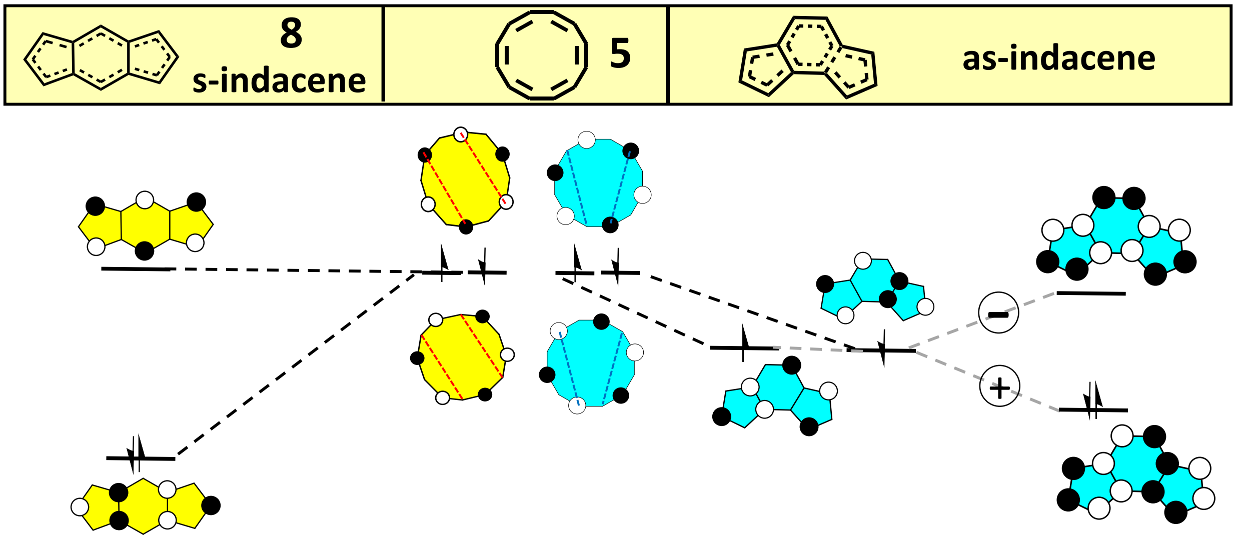

Figure S3. Evolution of the frontier molecular orbitals from planar [12]annulene (middle) to *s*-indacene (**8**, D_{2h}, left) and to *as*-indacene (C_{2v}, right).

4. References

[1] W. T. Borden, E. R. Davidson, Singlet-triplet energy separations in some hydrocarbon diradicals. *Ann. Rev. Phys. Chem.* **1979**, *30*, 125-153.

[2] K. Schulten, I. Ohmine, M. Karplus, Correlation effects in the spectra of polyenes. *J. Chem. Phys.* ***1976**, 64,* 4422-4441.

[3] Y. Shao, Z. Gan, E. Epifanovsky, A. T. B. Gilbert, M. Wormit, J. Kussmann, A. W. Lange, A. Behn, J. Deng, X. Feng, D. Ghosh, M. Goldey P. R. Horn, L. D. Jacobson, I. Kaliman, R. Z. Khaliullin, T. Kús, A. Landau, J. Liu, E. I. Proynov, Y. M. Rhee, R. M. Richard, M. A. Rohrdanz, R. P. Steele, E. J. Sundstrom, H. L. Woodcock III, P. M. Zimmerman, D. Zuev, B. Albrecht, E. Alguire, B. Austin, G. J. O. Beran, Y. A. Bernard, E. Berquist, K. Brandhorst, K. B. Bravaya, S. T. Brown, D. Casanova, C.-M. Chang, Y. Chen, S. H. Chien, K. D. Closser, D. L. Crittenden, M. Diedenhofen, R. A. DiStasio Jr., H. Dop, A. D. Dutoi, R. G. Edgar, S. Fatehi, L. Fusti-Molnar, A. Ghysels, A. Golubeva-Zadorozhnaya, J. Gomes, M. W. D. Hanson-Heine, P. H. P. Harbach, A. W. Hauser, E. G. Hohenstein, Z. C. Holden, T.-C. Jagau, H. Ji, B. Kaduk, K. Khistyaev, J. Kim, J. Kim, R. A. King, P. Klunzinger, D. Kosenkov, T. Kowalczyk, C. M. Krauter, K. U. Lao, A. Laurent, K. V. Lawler, S. V. Levchenko, C. Y. Lin, F. Liu, E. Livshits, R. C. Lochan, A. Luenser, P. Manohar, S. F. Manzer, S.-P. Mao, N. Mardirossian, A. V. Marenich, S. A. Maurer, N. J. Mayhall, C. M. Oana, R. Olivares-Amaya, D. P. O'Neill, J. A. Parkhill, T. M. Perrine, R. Peverati, P. A. Pieniazek, A. Prociuk, D. R. Rehn, E. Rosta, N. J. Russ, N. Sergueev, S. M. Sharada, S. Sharmaa, D. W. Small, A. Sodt, T. Stein, D. Stück, Y.-C. Su, A. J. W. Thom, T. Tsuchimochi, L. Vogt, O. Vydrov, T. Wang, M. A. Watson, J. Wenzel, A. White, C. F. Williams, V. Vanovschi, S. Yeganeh, S. R. Yost, Z.-Q. You, I. Y. Zhang, X. Zhang, Y. Zhou, B. R. Brooks, G. K. L. Chan, D. M. Chipman, C. J. Cramer, W. A. Goddard III, M. S. Gordon, W. J. Hehre, A. Klamt, H. F. Schaefer III, M. W. Schmidt, C. D. Sherrill, D. G. Truhlar, A. Warshel, X. Xua, A. Aspuru-Guzik, R. Baer, A. T. Bell, N. A. Besley, J.-D. Chai, A. Dreuw, B. D. Dunietz, T. R. Furlani, S. R. Gwaltney, C.-P. Hsu, Y. Jung, J. Kong, D. S. Lambrecht, W. Liang, C. Ochsenfeld, V. A. Rassolov, L. V. Slipchenko, J. E. Subotnik, T. Van Voorhis, J. M. Herbert, A. I. Krylov, P. M. W. Gill, M. Head-Gordon. Advances in molecular quantum chemistry contained in the Q-Chem 4 program package, Molecular Physics, **2014**, *113*, 184-215.

[4] T. Stuyver, B. Chen, T. Zen, P. Geerlings, F. De Proft, R. Hoffmann, Do Diradicals Behave Like Radicals? *Chem. Rev.* **2019**, *119*, 11291–11351.

[5] D. Doehnert, J. Koutecký, Occupation Numbers of Natural Orbitals as a Criterion for Biradical Character. Different Kinds of Biradicals, *J. Am. Chem. Soc*. **1980**, *102*, 1789–1796

[6] For a recent example for the use of y_o for the teranthene diradical molecule see A. Konishi, Y. Hirao, M. Nakano, A. Shimizu, E. Botek, B. Champagne, D. Shiomi, K. Sato, T. Takui, K. Matsumoto, H. Kurata, T. Kubo, Synthesis and characterization of teranthene: a singlet biradical polycyclic aromatic hydrocarbon having Kekulé structures, *J. Am. Chem. Soc*. **2010**,*132*, 11021–11023.

[7] Y. Carissan, D. Hagebaum-Reignier, N. Goudard, S. Humbel, HuLiS Program: Simple Hückel and LewIs embedded in Hückel theory, http://www.hulis.free.fr, **2008**.

[8] G. Schaftenaar, J. H. Noordik, Molden: a pre- and post-processing program for molecular and electronic structures, *J. Comput. Aided Mol. Design* **2000**, *14*, 123-134.

5. CARTESIAN COORDINATES OF OPTIMIZED GEOMETRIES

Compound 2

E_{tot}((U)B3LYP/6-31G(d))= -308.36753861a.u

C	0.00000000	0.70628300	0.00000000
C	-1.35345400	1.14188200	0.00000000
C	-2.18718100	0.00000000	0.00000000
C	-1.35345400	-1.14188200	0.00000000
C	0.00000000	-0.70628300	0.00000000
C	1.35345400	-1.14188200	0.00000000
C	2.18718100	0.00000000	0.00000000
C	1.35345400	1.14188200	0.00000000
H	1.70542200	2.16880000	0.00000000
H	3.26800400	0.00000000	0.00000000
H	1.70542200	-2.16880000	0.00000000
H	-1.70542200	-2.16880000	0.00000000
H	-3.26800400	0.00000000	0.00000000
H	-1.70542200	2.16880000	0.00000000

Compound 2-JT

E_{tot}((U)B3LYP/6-31G(d))= -308.37906391 a.u

C	-0.04725100	0.72824800	0.00000000
C	-1.47914600	1.08015500	0.00000000
C	-2.17003500	-0.08586400	0.00000000
C	-1.21037100	-1.23373100	0.00000000
C	0.04725100	-0.72824800	0.00000000
C	1.47914600	-1.08015500	0.00000000
C	2.17003500	0.08586400	0.00000000
C	1.21037100	1.23373100	0.00000000
H	1.51016700	2.27623300	0.00000000
H	3.24739600	0.19717900	0.00000000
H	1.89531500	-2.07930600	0.00000000
H	-1.51016700	-2.27623300	0.00000000
H	-3.24739600	-0.19717900	0.00000000
H	-1.89531500	2.07930600	0.00000000

Compound 3

E_{tot}((U)B3LYP/6-31G(d))= -615.72205268 a.u

C	1.89784600	1.08004500	0.00000000
C	0.61829300	1.71642300	0.00000000
C	-0.37053000	0.63602100	0.00000000
C	0.37052900	-0.63602000	0.00000000
C	1.70443400	-0.38415700	0.00000000
C	-1.70443500	0.38415600	0.00000000

C	-1.89784600	-1.08004600	0.00000000
C	-0.61829300	-1.71642300	0.00000000
C	3.05936500	1.84067100	0.00000000
C	2.95266400	3.24194500	0.00000000
C	1.70443400	3.86165100	0.00000000
C	0.52293400	3.09952100	0.00000000
C	-3.05936500	-1.84067100	0.00000000
C	-2.95266400	-3.24194600	0.00000000
C	-1.70443500	-3.86165100	0.00000000
C	-0.52293300	-3.09952000	0.00000000
H	2.51542100	-1.10399700	0.00000000
H	-2.51542300	1.10399500	0.00000000
H	4.03632600	1.36511000	0.00000000
H	3.85394900	3.84778500	0.00000000
H	1.64149000	4.94575600	0.00000000
H	-0.44522700	3.59217000	0.00000000
H	-4.03632500	-1.36511000	0.00000000
H	-3.85395000	-3.84778400	0.00000000
H	-1.64148700	-4.94575500	0.00000000
H	0.44522700	-3.59217100	0.00000000

Compound 4

E_{tot}((U)B3LYP/6-31G(d))= -615.68762385 a.u

C	0.00000000	1.36147600	-0.35642200
C	0.00000000	1.98158200	-1.61173400
C	0.00000000	3.37505800	-1.68095900
C	0.00000000	4.17808200	-0.52217500
C	0.00000000	3.59185800	0.73847900
C	0.00000000	2.19076000	0.84228300
C	0.00000000	1.33777700	1.98349900
C	0.00000000	0.00000000	1.50986900
C	0.00000000	0.00000000	0.08696800
C	0.00000000	-1.36147600	-0.35642200
C	0.00000000	-2.19076000	0.84228300
C	0.00000000	-1.33777700	1.98349900
C	0.00000000	-1.98158200	-1.61173400
C	0.00000000	-3.37505800	-1.68095900
C	0.00000000	-4.17808200	-0.52217500
C	0.00000000	-3.59185800	0.73847900
H	0.00000000	1.39169600	-2.52331300
H	0.00000000	3.85617600	-2.65522700
H	0.00000000	5.25899800	-0.62011200
H	0.00000000	4.20705100	1.63445600
H	0.00000000	1.66844300	3.01586100
H	0.00000000	-1.66844300	3.01586100
H	0.00000000	-1.39169600	-2.52331300
H	0.00000000	-3.85617600	-2.65522700
H	0.00000000	-5.25899800	-0.62011200
H	0.00000000	-4.20705100	1.63445600

Compound 6

E_{tot}((U)B3LYP/6-31G(d))= -463.21731316 a.u

C	-1.19742900	-1.51811000	0.00000000
C	-2.55458200	-1.22635100	0.00000000
C	-3.21943400	0.00000000	0.00000000
C	-2.55458200	1.22635100	0.00000000
C	-1.19742900	1.51811000	0.00000000
C	0.00000000	0.72503100	0.00000000
C	0.00000000	-0.72503100	0.00000000
C	1.19742900	1.51811000	0.00000000
C	2.55458200	1.22635100	0.00000000
C	3.21943400	0.00000000	0.00000000
C	2.55458200	-1.22635100	0.00000000
C	1.19742900	-1.51811000	0.00000000
H	-0.99216800	-2.58502000	0.00000000
H	-3.19334200	-2.10897100	0.00000000
H	-4.30396400	0.00000000	0.00000000
H	-3.19334200	2.10897100	0.00000000
H	-0.99216800	2.58502000	0.00000000
H	0.99216800	2.58502000	0.00000000
H	3.19334200	2.10897100	0.00000000
H	4.30396400	0.00000000	0.00000000
H	3.19334200	-2.10897100	0.00000000
H	0.99216800	-2.58502000	0.00000000

Compound 6 J-T

E_{tot}((U)B3LYP/6-31G(d))= -463.22292648 a.u

C	0.00009200	-0.72513300	-0.00021100
C	1.19701900	-1.51802200	0.00010800
C	2.55456600	-1.22629400	0.00065300
C	3.21919600	-0.00011300	0.00052700
C	2.55440500	1.22637400	-0.00048700
C	1.19719800	1.51812700	-0.00074300
C	-0.00009300	0.72513300	-0.00019800
C	-1.19701800	1.51802200	0.00022800
C	-2.55456600	1.22629400	0.00074700
C	-3.21919600	0.00011300	0.00032400
C	-2.55440600	-1.22637400	-0.00046900
C	-1.19719800	-1.51812700	-0.00055000
H	0.99182700	-2.58503700	0.00023800
H	3.19322200	-2.10898800	0.00118100
H	4.30382500	-0.00008900	0.00088700
H	3.19310300	2.10903900	-0.00095800
H	0.99202500	2.58514600	-0.00137800
H	-0.99182600	2.58503700	0.00048400

H	-3.19322200	2.10898800	0.00134200
H	-4.30382500	0.00009100	0.00059200
H	-3.19310300	-2.10903900	-0.00091100
H	-0.99202600	-2.58514700	-0.00105300

Compound 7

E_{tot}((U)B3LYP/6-31G(d))= -1075.44476050 a.u

C	1.56918300	-1.17841100	-0.00002400
C	1.28559600	-2.57269300	-0.00004200
C	0.00000900	-3.17102700	-0.00007600
C	-1.28557000	-2.57269800	-0.00001100
C	-1.56917200	-1.17841600	-0.00001700
C	-0.68866300	-0.00000600	-0.00006600
C	0.68866300	0.00000700	-0.00007000
C	-1.56918500	1.17841100	-0.00000800
C	-1.28559500	2.57269000	-0.00003300
C	-0.00001000	3.17102800	-0.00004700
C	1.28557200	2.57270000	0.00000000
C	1.56917000	1.17841600	0.00000000
H	0.00001400	-4.25824600	-0.00012600
H	-0.00001300	4.25824600	-0.00007200
C	-2.91081300	-0.73099800	0.00003300
C	-2.91081800	0.73098100	0.00001100
C	2.91081500	-0.73097700	-0.00000800
C	2.91081200	0.73099500	0.00003000
C	-2.41321000	-3.45043600	0.00005300
C	-2.41323100	3.45042100	-0.00004800
C	2.41323100	-3.45042200	-0.00006200
C	2.41320800	3.45043500	0.00006500
H	2.22370300	4.51992600	0.00007700
C	-3.71869500	-2.98959100	0.00010100
C	-3.98785500	-1.61347000	0.00009200
C	-3.98786800	1.61344900	0.00000700
C	-3.71872100	2.98956500	-0.00002600
C	3.71869800	2.98958800	0.00010500
C	3.98785500	1.61347100	0.00008000
C	3.98786600	-1.61344900	-0.00003100
C	3.71872300	-2.98956300	-0.00005700
H	-5.01036100	1.24913600	0.00004100
H	-4.53878700	3.70167700	-0.00002400
H	-2.22373000	4.51991200	-0.00007000
H	-5.01035100	-1.24916600	0.00013400
H	-4.53875800	-3.70170700	0.00015800
H	-2.22369300	-4.51992500	0.00006200
H	4.53875800	3.70170600	0.00016300
H	5.01035000	1.24916400	0.00011300
H	5.01035900	-1.24913200	-0.00000400
H	4.53879200	-3.70167000	-0.00005600
H	2.22374300	-4.51991500	-0.00008800

Compound 8

E_{tot}((U)B3LYP/6-31G(d))= -462.04054285 a.u

C	0.00000000	1.44057800	0.00000000
C	-1.20083800	0.72270900	0.00000000
C	-1.20083800	-0.72270900	0.00000000
C	0.00000000	-1.44057800	0.00000000
C	1.20083800	-0.72270900	0.00000000
C	1.20083800	0.72270900	0.00000000
C	2.56395000	-1.14034500	0.00000000
C	3.38489500	0.00000000	0.00000000
C	2.56395000	1.14034500	0.00000000
C	-2.56395000	1.14034500	0.00000000
C	-3.38489500	0.00000000	0.00000000
C	-2.56395000	-1.14034500	0.00000000
H	0.00000000	2.52890600	0.00000000
H	0.00000000	-2.52890600	0.00000000
H	2.90672400	2.16903300	0.00000000
H	-2.90672400	2.16903300	0.00000000
H	4.46736400	0.00000000	0.00000000
H	2.90672400	-2.16903300	0.00000000
H	-2.90672400	-2.16903300	0.00000000
H	-4.46736400	0.00000000	0.00000000

Compound 8-JT

E_{tot}((U)B3LYP/6-31G(d))= -462.04063324 a.u

C	0.00000000	0.00000000	1.44045600
C	0.00013100	1.20152900	0.72356200
C	0.00013100	1.20152900	-0.72356200
C	0.00000000	0.00000000	-1.44045600
C	-0.00013100	-1.20152900	-0.72356200
C	-0.00013100	-1.20152900	0.72356200
C	-0.00049200	-2.56207900	-1.14075500
C	-0.00006700	-3.38338700	0.00000000
C	-0.00049200	-2.56207900	1.14075500
C	0.00049200	2.56207900	1.14075500
C	0.00006700	3.38338700	0.00000000
C	0.00049200	2.56207900	-1.14075500
H	0.00000000	0.00000000	2.52889900
H	0.00000000	0.00000000	-2.52889900
H	-0.00045500	-2.90548800	2.16919600
H	0.00045500	2.90548800	2.16919600
H	0.00017200	-4.46561800	0.00000000
H	-0.00045500	-2.90548800	-2.16919600
H	0.00045500	2.90548800	-2.16919600
H	-0.00017200	4.46561800	0.00000000

Compound 9

E_{tot}((U)B3LYP/6-31G(d))= -462.04456598 a.u

C	0.75456500	0.71192700	-0.00000100
C	0.75456500	-0.71192700	-0.00000100
C	1.91524100	-1.44547400	0.00000000
C	3.12109600	-0.69495500	0.00000000
C	3.12109600	0.69495500	0.00000000
C	1.91524200	1.44547400	0.00000000
C	-0.75456500	-0.71192700	0.00000200
C	-0.75456500	0.71192600	0.00000200
C	-1.91524200	1.44547400	0.00000000
C	-3.12109500	0.69495600	-0.00000100
C	-3.12109600	-0.69495500	0.00000000
C	-1.91524200	-1.44547300	0.00000000
H	1.93557600	-2.53085500	0.00000100
H	4.07035900	-1.22285400	0.00000000
H	4.07035900	1.22285300	0.00000000
H	1.93557600	2.53085500	0.00000100
H	-1.93557400	2.53085500	-0.00000200
H	-4.07035900	1.22285300	0.00000000
H	-4.07035900	-1.22285300	-0.00000100
H	-1.93557600	-2.53085500	-0.00000100

Compound 10

E_{tot}((U)B3LYP/6-31G(d))= -769.36873813 a.u

C	0.41995500	-1.38854800	-0.00009300
C	-0.96754500	-1.01714500	-0.00009300
C	-1.35688500	0.40035700	-0.00010600
C	-0.41997500	1.38844200	-0.00009200
C	0.96750100	1.01699500	-0.00009300
C	1.35689100	-0.40050300	-0.00010700
C	2.10687200	1.78878300	0.00002200
C	3.27321400	0.92114300	0.00004000
C	2.82391200	-0.43196700	-0.00003800
C	-2.10696800	-1.78886000	0.00002200
C	-3.27327900	-0.92115800	0.00004200
C	-2.82386700	0.43191900	-0.00003700
C	-4.63865800	-1.21067800	0.00012300
C	-5.55243100	-0.15174900	0.00012300
C	-5.10943600	1.17550100	0.00003200
C	-3.74031600	1.47623500	-0.00005500
C	4.63856600	1.21078100	0.00012300
C	5.55243300	0.15193200	0.00012500
C	5.10955400	-1.17536000	0.00003300
C	3.74046400	-1.47619400	-0.00005600
H	0.69673200	-2.44016800	-0.00006900
H	-0.69676400	2.44005400	-0.00006700
H	2.14138700	2.87259300	0.00003600

H	-2.14157100	-2.87267100	0.00004100
H	-4.98764100	-2.23965100	0.00018200
H	-6.61808400	-0.36078800	0.00018700
H	-5.83564300	1.98286200	0.00003600
H	-3.40923200	2.51123900	-0.00013100
H	4.98747500	2.23977800	0.00018400
H	6.61807000	0.36106200	0.00019000
H	5.83584600	-1.98264100	0.00003800
H	3.40942400	-2.51121500	-0.00013200

Compound 11

E_{tot}((U)B3LYP/6-31G(d))= -769.35070180a.u

C	0.00007700	2.08766300	0.00016900
C	-1.20073600	1.36744800	0.00002100
C	-1.19869400	-0.07387600	0.00003500
C	-0.00001300	-0.78760400	0.00012400
C	1.19863700	-0.07380900	0.00006800
C	1.20079700	1.36754400	0.00010600
C	2.58549000	-0.49953300	-0.00006900
C	3.40814500	0.68505300	0.00007700
C	2.55715600	1.82098500	0.00011000
C	-2.55694900	1.82100800	-0.00024500
C	-3.40817100	0.68504700	-0.00016600
C	-2.58546100	-0.49967900	0.00007800
C	-4.81262000	0.57156300	-0.00022100
C	-5.38587700	-0.69292700	-0.00006000
C	-4.57817800	-1.84835200	0.00016300
C	-3.18501500	-1.76022700	0.00022300
C	3.18488800	-1.76027300	-0.00019200
C	4.57801000	-1.84849500	-0.00019600
C	5.38583100	-0.69300900	-0.00007700
C	4.81274500	0.57147600	0.00009000
H	-0.00002800	3.17513000	0.00014800
H	-0.00009200	-1.87512400	0.00013900
H	2.87742700	2.85649300	0.00017000
H	-2.87736200	2.85646900	-0.00038900
H	-5.43542200	1.46200100	-0.00037500
H	-6.46662700	-0.79764800	-0.00010600
H	-5.05092100	-2.82634400	0.00026900
H	-2.58228400	-2.66428500	0.00034400
H	2.58205400	-2.66424700	-0.00024600
H	5.05070500	-2.82650600	-0.00027100
H	6.46657500	-0.79782500	-0.00009600
H	5.43558900	1.46187100	0.00017300

Compound 12

E_{tot}((U)B3LYP/6-31G(d))= -616.87891428 a.u

C	-2.41402600	1.55765400	-0.00000100
C	-3.76394100	1.24799100	0.00003300
C	-4.39945600	-0.00001000	0.00004000
C	-3.76394800	-1.24798600	-0.00003800
C	-2.41400900	-1.55764900	-0.00007500
C	-1.25066600	-0.72472200	0.00003500
C	-1.25065500	0.72472000	0.00004700
C	2.41402300	-1.55765600	0.00003700
C	3.76393900	-1.24799000	0.00004200
C	4.39945500	0.00000900	0.00000200
C	3.76394800	1.24798800	-0.00008300
C	2.41401300	1.55765100	-0.00009900
H	-2.18724400	2.62153400	-0.00002600
H	-4.42808500	2.11078800	0.00005900
H	-5.48544300	-0.00000600	0.00009100
H	-4.42808200	-2.11079000	-0.00007200
H	-2.18723000	-2.62152900	-0.00015700
H	2.18723800	-2.62153400	0.00004800
H	4.42808300	-2.11078700	0.00007600
H	5.48544200	0.00000400	0.00002600
H	4.42808500	2.11079000	-0.00013100
H	2.18723200	2.62152900	-0.00017100
C	1.25065600	-0.72472000	0.00005000
C	1.25066500	0.72472100	0.00001500
C	0.00000900	-1.36456600	0.00003200
C	-0.00000700	1.36456700	0.00001300
H	0.00000600	-2.45128500	-0.00000100
H	-0.00000700	2.45128500	-0.00003500

Compound 13

E_{tot}((U)B3LYP/6-31G(d))= -1381.54520751 a.u

C	-2.60122300	-1.35096300	-0.00000600
C	-3.91808600	-0.74015500	0.00000500
C	-4.26281300	0.65113900	0.00009800
C	-3.49990000	1.84984800	0.00002900
C	-2.09011600	1.89084700	0.00000000
C	-1.16109200	0.78558500	0.00001700
C	-1.35236200	-0.61100600	0.00002300
C	2.60122000	1.35096300	-0.00006000
C	3.91808200	0.74015700	-0.00003200
C	4.26281400	-0.65113600	-0.00001600
C	3.49990100	-1.84984700	-0.00001400
C	2.09011700	-1.89084900	-0.00000600
H	-5.33708800	0.82189500	0.00021300
H	5.33708900	-0.82189100	0.00002600
C	1.35236000	0.61100400	-0.00004300

C	1.16109200	-0.78558900	0.00000500
C	0.14178500	1.33452100	-0.00002500
C	-0.14178500	-1.33452500	0.00003800
C	-1.36479400	3.11253400	-0.00002500
C	0.06585400	2.76580900	-0.00001900
C	-0.06585700	-2.76581300	0.00003700
C	1.36479700	-3.11254000	-0.00000700
C	-2.50434900	-2.78701100	-0.00001800
C	-5.03964600	-1.61242800	-0.00001800
C	-4.15682900	3.11672800	0.00007900
C	2.50434400	2.78701200	-0.00001700
C	5.03964100	1.61242900	0.00001400
C	4.15683700	-3.11672400	-0.00000300
C	-3.44800700	4.30977600	0.00006500
C	-2.03997600	4.32856100	-0.00003000
C	1.23104500	3.46995000	-0.00000700
C	3.67159600	3.58312400	0.00003600
C	4.92645700	2.99605600	0.00004600
H	-5.24320300	3.13901800	0.00012800
H	-3.99229800	5.24962400	0.00008800
H	-1.50373200	5.27272400	-0.00003100
H	1.24398800	4.55694300	-0.00002600
H	3.56702300	4.66401400	0.00006700
H	5.82113500	3.61147000	0.00011700
H	6.02894700	1.16492600	0.00001400
C	-4.92646300	-2.99605600	-0.00006900
C	-3.67160000	-3.58312200	-0.00006900
C	-1.23104800	-3.46995100	-0.00000100
H	-6.02895100	-1.16492400	-0.00000500
H	-5.82114000	-3.61147100	-0.00010900
H	-3.56702700	-4.66401200	-0.00008000
H	-1.24399500	-4.55694300	-0.00001100
C	2.03998600	-4.32856000	-0.00004400
C	3.44801500	-4.30977100	-0.00002000
H	1.50375300	-5.27273000	-0.00003400
H	3.99230700	-5.24962000	-0.00003500
H	5.24321200	-3.13900200	0.00002400

Compound 14

E_{tot}((U)B3LYP/6-31G(d))= -500.82476793 a.u

C	0.93037700	-1.08729700	-0.00061600
C	0.00000200	-0.00000500	-0.00028400
C	0.47644100	1.34937300	-0.00061400
C	-0.46776200	2.40786800	0.00018000
C	-1.83483300	2.14392300	0.00046300
C	-2.30679300	0.83417600	0.00015300
C	-1.85141000	-1.60902000	0.00018100
C	-0.93928600	-2.66096200	0.00047000
C	0.43097300	-2.41482800	0.00015600

C	-1.40680900	-0.26207200	-0.00061200
C	2.31916700	-0.79884900	0.00017400
C	2.77411400	0.51704600	0.00046500
C	1.87581900	1.58064800	0.00016100
H	-0.10802700	3.43304400	-0.00147200
H	-2.54102400	2.96930400	0.00030900
H	-3.37518000	0.63686600	0.00060300
H	-2.91911100	-1.81005400	-0.00147600
H	-1.30098500	-3.68523500	0.00031700
H	1.13601800	-3.24144500	0.00060900
H	3.02713100	-1.62297600	-0.00148200
H	3.84201000	0.71594000	0.00031400
H	2.23915800	2.60454800	0.00062200

Compound 16

E_{tot}((U)B3LYP/6-31G(d))= -730.71132129 a.u

C	1.23412500	-1.54502700	-0.00007200
C	1.23538200	-0.10860000	-0.00010100
C	2.46922300	0.60386300	-0.00008500
C	3.68219800	-0.12258700	0.00006800
C	3.67683000	-1.51652500	0.00017800
C	2.47825900	-2.22341600	0.00008400
C	-0.00000100	-2.22929400	-0.00013500
C	-1.23411600	-1.54502300	-0.00010300
C	-1.23538700	-0.10860200	-0.00003200
C	0.00000100	0.60290600	-0.00004400
C	-2.47826000	-2.22341800	-0.00002400
C	-3.67681700	-1.51654300	0.00009200
C	-3.68219200	-0.12259700	0.00007200
C	-2.46923800	0.60385700	-0.00000600
C	-2.43530500	2.03308700	0.00000300
C	-1.24713200	2.71582300	0.00002600
C	0.00000500	2.02896800	0.00000800
C	1.24714300	2.71580700	-0.00001500
C	2.43528300	2.03305600	-0.00005000
H	4.62282000	0.42099500	0.00005500
H	4.61887800	-2.05714100	0.00032400
H	2.48369100	-3.30983100	0.00006900
H	0.00000200	-3.31641200	-0.00020100
H	-2.48369700	-3.30983600	-0.00012400
H	-4.61886600	-2.05716200	0.00022400
H	-4.62282300	0.42096200	0.00020600
H	-3.37759100	2.57433400	0.00023100
H	-1.24184700	3.80270100	0.00008700
H	1.24185500	3.80268400	0.00003100
H	3.37757400	2.57430400	-0.00009500

Compound 17

E_{tot}((U)B3LYP/6-31G(d))= -844.32708138 a.u

C	-0.86209700	-1.07601100	0.55288600
C	0.52003100	-1.27018600	0.56336700
C	1.41465300	-0.19688800	0.50194400
C	0.86210700	1.07605600	0.55298700
C	-0.52003200	1.27020900	0.56344800
C	-1.41467000	0.19691600	0.50195500
C	-2.74227400	0.38737300	0.01277400
C	-3.50488500	-0.84713000	-0.23429200
C	-2.93786700	-2.09734900	-0.21229200
C	-1.51816900	-2.27281600	0.08601600
C	-0.52287900	-3.21526100	-0.16275900
C	0.80184900	-2.57160500	0.07564800
C	2.11656700	-2.77590600	-0.33694600
C	3.07575100	-1.70010800	-0.33733900
C	2.74223700	-0.38734600	0.01267800
C	3.50486600	0.84708500	-0.23432200
C	2.93792600	2.09733200	-0.21218600
C	1.51818700	2.27285100	0.08609900
C	0.52298800	3.21523400	-0.16291000
C	-0.80190000	2.57158500	0.07560200
C	-2.11658700	2.77593300	-0.33695200
C	-3.07583100	1.70006900	-0.33726500
H	-4.55122500	-0.75309000	-0.51326000
H	-3.55142200	-2.95555700	-0.47342200
H	-0.66258200	-4.20922900	-0.57108800
H	2.43345500	-3.72510700	-0.76315000
H	4.06519200	-1.91442300	-0.73338100
H	4.55121500	0.75304900	-0.51327700
H	3.55152700	2.95557600	-0.47308200
H	0.66273700	4.20906200	-0.57155800
H	-2.43347900	3.72507200	-0.76328800
H	-4.06524400	1.91440900	-0.73335000

Compound 18

E_{tot}((U)B3LYP/6-31G(d))= -845.57746147 a.u

C	3.76874400	0.81015200	0.00042900
C	4.88020300	0.00319500	0.00008800
C	4.70710500	-1.42313400	-0.00000700
C	3.46698400	-2.02011100	-0.00002500
C	2.28023800	-1.21156500	-0.00004300
C	2.48455500	0.18339700	0.00035500
C	0.95116100	-1.65103400	-0.00023900
C	-0.12295600	-0.73057700	-0.00038700
C	0.12295800	0.73052500	-0.00024900
C	1.47866800	1.14691000	-0.00004800
C	-1.47864200	-1.14686600	-0.00060800

C	-2.48451400	-0.18340100	-0.00097100
C	-2.28018500	1.21156000	-0.00045300
C	-0.95111800	1.65098700	-0.00021800
C	-3.76873200	-0.81018000	-0.00019600
C	-4.88013900	-0.00326900	0.00042500
C	-4.70712500	1.42305600	0.00027100
C	-3.46709000	2.02013300	0.00000000
C	2.15830800	2.45188100	0.00004500
C	3.51000300	2.25483200	0.00015100
C	-2.15836600	-2.45178800	0.00039400
C	-3.51003600	-2.25468700	0.00061000
H	5.88696200	0.41298900	-0.00025600
H	5.59585600	-2.04749100	-0.00025900
H	3.38114700	-3.10326200	0.00042200
H	0.72766400	-2.71414200	-0.00060900
H	-0.72757000	2.71408000	0.00017300
H	-5.88688600	-0.41306700	0.00089400
H	-5.59589900	2.04736800	0.00049900
H	-3.38137400	3.10329200	0.00044800
H	1.66415700	3.41575700	0.00023900
H	4.26885300	3.02722100	0.00101100
H	-1.66424900	-3.41568700	0.00046800
H	-4.26880400	-3.02716800	0.00101200

Compound 19

E_{tot}((U)B3LYP/6-31G(d))= -1266.67390999 a.u

C	-1.85608300	-3.28769200	0.00015100
C	-2.07858200	-4.69033600	0.00020100
C	-1.00949500	-5.58229900	0.00002800
C	0.30426200	-5.12121400	-0.00020900
C	0.58710500	-3.72949200	-0.00022500
C	-0.50715500	-2.80449100	-0.00002700
C	1.90671000	-3.23182300	-0.00038400
C	2.18409500	-1.84900100	-0.00024300
C	1.08534400	-0.91882400	-0.00021100
C	-0.25335900	-1.40106400	-0.00009700
C	3.50223100	-1.34717400	0.00008700
C	3.77528900	0.03642000	0.00008700
C	2.68237900	0.96304300	0.00005400
C	1.34005300	0.48113500	-0.00010600
C	5.10128600	0.54504000	0.00022900
C	5.33928300	1.91686500	0.00033300
C	4.28310800	2.82411700	0.00011500
C	2.93636500	2.37320400	-0.00002300
C	-1.33840400	-0.48051000	-0.00001400
C	-2.69333300	-0.96698700	0.00022100
C	-2.91781300	-2.35941200	0.00034500
C	0.25308500	1.39935300	-0.00019100
C	-1.08668500	0.91996900	-0.00008200

C	1.84559300	3.26721200	-0.00011900
C	0.50925800	2.81599000	-0.00009900
C	-2.17519200	1.84146500	-0.00008100
C	-3.52345600	1.35626000	0.00006000
C	-3.75225300	-0.03532800	0.00034400
C	-0.58443500	3.70665300	0.00002700
C	-1.91923700	3.25126600	0.00004800
C	-3.02275700	4.14524700	-0.00012300
C	-4.32983400	3.66534000	-0.00032100
C	-4.58738100	2.29709400	-0.00001200
H	-3.10022600	-5.05938400	0.00035800
H	-1.20278500	-6.65110700	0.00008000
H	1.13195500	-5.82468600	-0.00037200
H	2.73564800	-3.93501100	-0.00066600
H	4.33256300	-2.04871900	0.00034900
H	5.93168100	-0.15524500	0.00008800
H	6.36154000	2.28390600	0.00047800
H	4.47857000	3.89263900	0.00014800
H	-3.94052700	-2.72775200	0.00051800
H	2.04013700	4.33668700	0.00002100
H	-4.77569200	-0.40164600	0.00063600
H	-0.39200200	4.77650400	-0.00010100
H	-2.83151900	5.21453000	0.00042900
H	-5.15883300	4.36711200	-0.00045300
H	-5.61044600	1.93201100	-0.00007600

Compound 20

E_{tot}((U)B3LYP/6-31G(d))= -1000.45872758 a.u

C	6.17212800	-1.40983800	0.00000000
C	7.34923000	-0.71575900	0.00000200
C	7.34923000	0.71575900	0.00000000
C	6.17212800	1.40983900	0.00000100
C	4.91055200	0.72719600	0.00000000
C	4.91055200	-0.72719600	0.00000000
C	3.69798600	1.40817900	0.00000000
C	2.45706900	0.72944000	0.00000000
C	2.45706900	-0.72944000	-0.00000100
C	3.69798600	-1.40818000	0.00000000
C	1.23196600	1.40979000	-0.00000100
C	0.00000000	0.73065400	-0.00000100
C	0.00000000	-0.73065400	-0.00000100
C	1.23196600	-1.40979000	-0.00000100
C	-1.23196600	1.40979000	-0.00000100
C	-2.45706900	0.72944000	0.00000000
C	-2.45706900	-0.72944000	-0.00000100
C	-1.23196600	-1.40979000	-0.00000100
C	-3.69798500	1.40817900	0.00000000
C	-4.91055300	0.72719600	0.00000000
C	-4.91055200	-0.72719600	0.00000000

C	-3.69798600	-1.40818000	0.00000000
C	-6.17212800	1.40983800	0.00000000
C	-7.34923000	0.71575900	0.00000000
C	-7.34923000	-0.71575900	0.00000200
C	-6.17212700	-1.40983800	0.00000000
H	6.17049200	-2.49670700	-0.00000100
H	8.29637800	-1.24704300	0.00000300
H	8.29637800	1.24704300	0.00000200
H	6.17049200	2.49670700	0.00000200
H	3.69869100	2.49573700	0.00000000
H	3.69869100	-2.49573700	0.00000000
H	1.23242400	2.49721700	-0.00000100
H	1.23242400	-2.49721700	-0.00000100
H	-1.23242400	2.49721700	-0.00000100
H	-1.23242300	-2.49721700	-0.00000100
H	-3.69869100	2.49573700	0.00000000
H	-3.69869200	-2.49573700	0.00000000
H	-6.17049200	2.49670700	0.00000200
H	-8.29637700	1.24704300	0.00000200
H	-8.29637700	-1.24704300	0.00000300
H	-6.17049200	-2.49670700	-0.00000100

Compound 21

E_{tot}((U)B3LYP/6-31G(d))= -1151.77258969 a.u

C	4.96257700	1.21138700	-0.00012300
C	3.55415600	1.24057500	0.00010200
C	2.83886500	0.00001700	0.00010100
C	3.55418700	-1.24055100	-0.00008500
C	4.96249800	-1.21141100	-0.00035000
C	5.65053800	0.00002700	-0.00036900
C	2.81640000	2.45957000	0.00018800
C	1.43578300	2.47966400	0.00009500
C	0.70469200	1.23401500	0.00019600
C	1.41837300	0.00002300	0.00006700
C	0.70464500	-1.23401000	0.00013200
C	1.43577500	-2.47970200	0.00010300
C	2.81636500	-2.45959400	-0.00001300
C	-0.70471600	1.23402000	0.00003200
C	-1.41839000	0.00000900	0.00008600
C	-0.70464300	-1.23400100	0.00002200
C	0.67785900	3.71115300	-0.00002600
C	-0.67784500	3.71114800	-0.00008400
C	-1.43580400	2.47969400	0.00013700
C	-1.43578300	-2.47969600	0.00006400
C	-0.67784600	-3.71117600	0.00019600
C	0.67784000	-3.71118000	0.00027400
C	-2.83886900	0.00002700	0.00005200
C	-3.55418500	-1.24055500	-0.00002400
C	-2.81637800	-2.45958600	-0.00010200

C	-2.81643800	2.45955200	0.00014300
C	-3.55411900	1.24057900	0.00008100
C	-4.96251500	1.21138100	-0.00023100
C	-5.65051900	0.00004900	-0.00023400
C	-4.96252100	-1.21142800	-0.00027500
H	5.50986900	2.14994900	-0.00011900
H	5.50985500	-2.14993100	-0.00053800
H	6.73664200	-0.00002800	-0.00062400
H	3.36469300	3.39863900	0.00030100
H	3.36468900	-3.39864500	-0.00000400
H	1.22754300	4.64861100	0.00018300
H	-1.22751400	4.64863100	0.00005600
H	-1.22758100	-4.64861500	0.00033700
H	1.22756800	-4.64862100	0.00050300
H	-3.36469400	-3.39864100	-0.00025500
H	-3.36472500	3.39862600	0.00030300
H	-5.50980800	2.14994100	-0.00039200
H	-6.73662100	0.00008600	-0.00046600
H	-5.50978900	-2.14999300	-0.00021600
